# CryoFIB milling large tissue samples for cryo-electron tomography

**DOI:** 10.1038/s41598-023-32716-z

**Published:** 2023-04-11

**Authors:** Sihan Wang, Heng Zhou, Wei Chen, Yifeng Jiang, Xuzhen Yan, Hong You, Xueming Li

**Affiliations:** 1 Key Laboratory for Protein Sciences of Ministry of Education, School of Life Sciences, Tsinghua University, Beijing, 100084 China; 2 School of Life Sciences, Tsinghua University, Beijing, 100084 China; 3 Tsinghua-Peking Joint Center for Life Sciences, Beijing, 100084 China; 4 Beijing Frontier Research Center for Biological Structure, Beijing, 100084 China; 5 Advanced Innovation Center for Structural Biology, Beijing, 100084 China; 6 Liver Research Center, Beijing Friendship Hospital, Capital Medical University, Beijing, 100050 China; 7 ZEISS Microscopy Customer Center, Beijing Laboratory, Beijing, 100088 China; 8 Beijing Key Laboratory of Translational Medicine in Liver Cirrhosis, National Clinical Research Center of Digestive Diseases, Beijing, 100050 China

**Keywords:** Cryoelectron microscopy, Cryoelectron tomography

## Abstract

Cryo-electron tomography (cryoET) is a powerful tool for exploring the molecular structure of large organisms. However, technical challenges still limit cryoET applications on large samples. In particular, localization and cutting out objects of interest from a large tissue sample are still difficult steps. In this study, we report a sample thinning strategy and workflow for tissue samples based on cryo-focused ion beam (cryoFIB) milling. This workflow provides a full solution for isolating objects of interest by starting from a millimeter-sized tissue sample and ending with hundred-nanometer-thin lamellae. The workflow involves sample fixation, pre-sectioning, a two-step milling strategy, and localization of the object of interest using cellular secondary electron imaging (CSEI). The milling strategy consists of two steps, a coarse milling step to improve the milling efficiency, followed by a fine milling step. The two-step milling creates a furrow–ridge structure with an additional conductive Pt layer to reduce the beam-induced charging issue. CSEI is highlighted in the workflow, which provides on-the-fly localization during cryoFIB milling. Tests of the complete workflow were conducted to demonstrate the high efficiency and high feasibility of the proposed method.

## Introduction

Cryo-electron tomography (cryoET) is a rapidly developing and popular technique that enables the direct study of high-resolution structures of biological macromolecules and their interactions in cells and tissues in situ^1,2^. However, due to the strong interactions between incident electrons and biological material, frozen-hydrated biological samples must be sliced into thin lamellae, typically not exceeding 200–300 nm thick^3^, ahead of cryoET imaging. Finding and cutting out a thin lamella containing the objects of interest from a millimeter-size tissue sample is one of the major challenges of cryoET, and effectively limits the application of this technology on large tissue samples.

Cryo-focused ion beam (cryoFIB) milling^4,5^ is a promising technique for cryoET sample preparation. In contrast to conventional cryo-ultramicrotomy using a diamond knife^6^, cryoFIB can avoid mechanical damage to the sample during the sectioning process. However, the ion beam has very low efficiency in removing large volumes. Consequently, cryoFIB is mostly used for samples with an initial thickness of several micrometers^2^. To improve the efficiency and enable cryoFIB to be used on large tissue samples, attempts have been made to combine cryo-ultramicrotomy with cryoFIB. For example, Hayles et al. froze cells in a copper tube using high-pressure freezing (HPF), then trimmed the tube using cryo-ultramicrotomy to a specific shape with suitable thickness for cryoFIB milling^7^. Zhang et al.^8^ froze a large tissue sample in a special cryo-carrier and subsequently trimmed the cryo-carrier together with the sample using cryo-ultramicrotomy to achieve an initial thickness of 20 μm for further cryoFIB milling. A technique called cryo-lift out used a cryo-gripper to extract a small volume from a large sample with the assistance of cryoFIB and then milled using the conventional cryoFIB method^9^. While these methods can be applied to large tissue samples, the complicated operational procedures required are a major disadvantage as only well-trained users can successfully apply these methods, and total processing times are counted in days.

Another key issue involves the localization of objects of interest before or during the cryoFIB process. The field of view of cryoET is limited within a thin lamella with micrometer width. Localization for the accurate three-dimensional (3D) position of such a small volume within a large bulky sample is required. Cryo-correlative light and electron microscopy (cryoCLEM)^10,11^ is a popular solution for this problem, which uses sophisticated fluorescence microscopes, primarily confocal and super-resolution fluorescence microscopes, to assist cryoFIB milling and cryoET analysis. In most applications to date, cryoCLEM has been performed outside the cryoFIB instrument, and hence is unable to achieve on-the-fly localization. Attempts to enable on-the-fly localization by integrating a fluorescence microscope into the cryoFIB instrument^12^ have been significantly limited by the poor resolution of fluorescence imaging due to the long working distance of the optical lens and limited installation space inside the cryoFIB instrument. Meanwhile, since the axial resolution of cryoCLEM is usually much lower than the lateral resolution, localization in the axial direction is difficult to accomplish during the milling^13^. Cellular secondary electron imaging (CSEI) provides a new option, which relies on the imaging capability of the secondary electrons of a scanning electron microscope (SEM) and enables high-contrast imaging of the cellular contents on a flat surface created by cryoFIB milling. Because the secondary electron imaging is a fundamental function of a cryoFIB instrument, CSEI does not require any additional instruments. In reports of cryoFIB-SEM block-face imaging^14–16^ and our accompanying work^17^, CSEI allows direct visualization of the ultrastructures in cells. Therefore, we hypothesized that CSEI has the potential to enable on-the-fly localization and assist cryoFIB milling for large tissue samples.

In this study, we report a cryoFIB milling method and workflow for frozen-hydrated tissue samples. This method allows for high-efficiency milling of large frozen-hydrated tissue samples with on-the-fly localization of objects of interest, without the need for extra equipments. This workflow covers all sample preparation steps, including fixation, pre-sectioning, CSEI localization, and coarse and fine milling. Additionally, the charging issue commonly observed in large tissue lamella is remarkably weakened by virtue of a furrow–ridge structure prepared in the fine milling step. Finally, an example of the complete workflow is illustrated by targeting collagen fibrils in mouse liver tissue.

## Results

### Overall workflow for high-efficiency milling of large tissue samples

**Figure 1:**
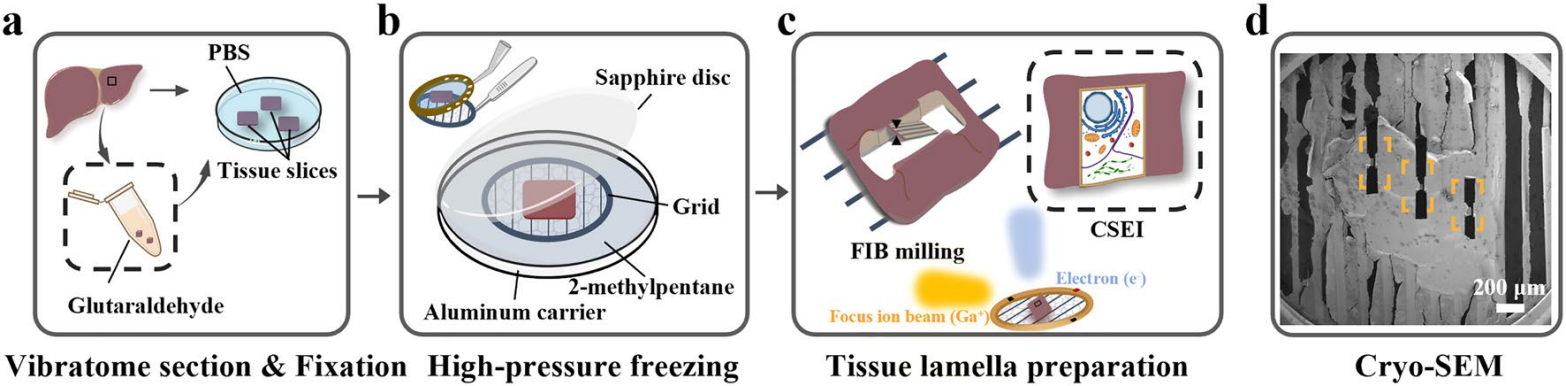
Schematic illustration of the overall workflow. (**a**) Chemical fixation and pre-sectioning starting from a large tissue. (**b**) High-pressure freezing (HPF) on a grid with parallel bars using 2-methylpentane. A Perfect Loop (Diatome, DZ8) is used to transfer the sliced sample to the grid. (**c**) CryoFIB milling with CSEI-based localization. The incident ion beam should be approximately along the direction of the grid bars in order to limit the milling within the space between neighoring bars. (**d**) Typical SEM image of the sample with multiple lamellae (yellow boxes) prepared on one tissue block.

The initial size of tissue samples is often > 1 mm, whereas the workable sample size for HPF and cryoFIB is much smaller. The gap of size requirement among these processing steps and size-reducing efficiency should be carefully considered. The optimal thickness for HPF must be ≤ 200 μm^18^ to ensure that the sample is fully vitrified. The initial thickness for cryoFIB determines the milling time, which increases significantly with the initial thickness; 0.5–1.5 h are required for samples thinner than 10 μm^19,20^, and several hours^9,21^ or even days^22^ may be required for multicellular organisms that are tens of microns thick. In addition, the ion beam current is a key factor that determines the milling speed. For example, if cutting a window on a sample takes a few minutes using nanoampere(nA)-level current, many hours would be required using picoampere(pA)-level current. Combining these analyses, we designed a workflow to achieve high-efficiency milling (Fig. 1).

The first step is the pre-sectioning, which uses a mechanical method to slice the initial sample to a workable thickness for HPF and cryoFIB milling (Fig. 1a). A cuboid with an edge length of 1–3 mm (smaller than a standard 3-mm grid) is cut out of the original bulky tissue and used as the initial sample for pre-sectioning. Chemical fixation, while optional, is often used ahead of pre-sectioning to minimize autolysis induced by the cellular contents from broken cells^23^ (Supplementary Fig. 1a,b). Additionally, fixation can increase the hardness of the tissue samples, thereby improving the accuracy of mechanical sectioning (discussed later).

The second step involves loading the sample to a grid and performing HPF (Fig. 1b). 2-methylpentane, which sublimes at − 150 °C under high vacuum, is the preferred cryo-protectant for HPF as it can be easily removed by warming after HPF^22^. We recommend using a grid with parallel bars (Fig. 1b), which lacks grid bars in one direction, to avoid obstructing the ion and electron beam during cryoFIB milling and subsequent data collection of tilt series.

The third step involves multiple platinum (Pt) coatings and a two-step milling strategy composed of coarse and fine milling sub-steps (Fig. 1c). The multiple Pt coating includes an organometallic Pt deposition to protect the sample from ion beam radiation damage (Supplementary Protocol D9) and two sputter coating to improve the surface electric conductivity (Supplementary Protocol D10 and E14). Coarse milling is performed to rapidly remove large sample volume using a high ion current at the nA level, while fine milling is used to generate the final lamella with a special lamella structure (discussed later). CSEI is used in the beginning of the coarse milling to localize the milling targets. With this milling strategy, the preparation of a tissue lamella as large as 20 × 50 μm typically requires only 6 h (Supplementary Tables 1 and 2). While the total milling time is longer than that for a thin sample, the larger lamella size contributed more cryoET tomograms per single lamella (Supplementary Tables 3 and 4).

This workflow should be compatible with most cryoFIB instruments. In this work, CSEI-based localization was performed on a Crossbean 550 (ZEISS Microscopy) for high-contrast CSEI, while further cryoFIB milling was performed on a Helios (FEI Company) for high-quality milling. Two cryoFIB instruments were involved here in order to utilize the advantages of different instruments. Finishing the workflow on a single instrument is still recommended for better efficiency.

### Chemical fixation and pre-sectioning

The pre-sectioning step was used to generate an initial workable thickness for HPF and cryoFIB milling. We recommend the use of a vibratome, which ensures the reproducibility of the sectioning process. The recommended section thickness is 20–80 μm; thinner sections require less time for subsequent cryoFIB milling.

Pre-sectioning often failed for very soft tissue samples; therefore, increasing the sample hardness by chemical fixation became necessary. We tested the success rate following different fixation times using 2.5% glutaraldehyde on mouse liver samples at room temperature (Supplementary Fig. 1c). Longer fixation time allows more glutaraldehyde to diffuse into the sample, resulting in a less broken central region; in the samples with shorter fixation time, the central region is often broken or appears brighter (Supplementary Fig. 1c). Note that even though the central region is broken, the surrounding region is often still large enough for cryoFIB milling. Therefore, a fixation time of 10–30 min was used in subsequent experiments.

Notably, we observed that the actual sample thickness was often larger than the pre-set thickness on the vibratome (Supplementary Fig. 1d,e). Additionally, the thickness is sometimes uneven in different areas of the same sample (Supplementary Fig. 1f). These issues should be considered when designing the experiments.

### On-the-fly localization using CSEI

Secondary electron imaging is a basic function built into the cryoFIB instrument and is used to observe the surface topography and assist with cryoFIB milling. Under some settings, secondary electron imaging can capture cellular contrast on a flat surface created by cryoFIB milling. In our accompanying work^17^, we introduced CSEI to facilitate on-the-fly localization during cryoFIB milling. The imaging mechanism of CSEI is related to the secondary electron emission efficiency, the electrical conductivity of the bulk sample, and the surface charge under primary electron radiation. CSEI captures different contrast in cellular contents with different water content, membranes, and protein condensates. Importantly, CSEI can stably and rapidly display cellular features on the surface created by variant ion beam currents.

**Figure 2:**
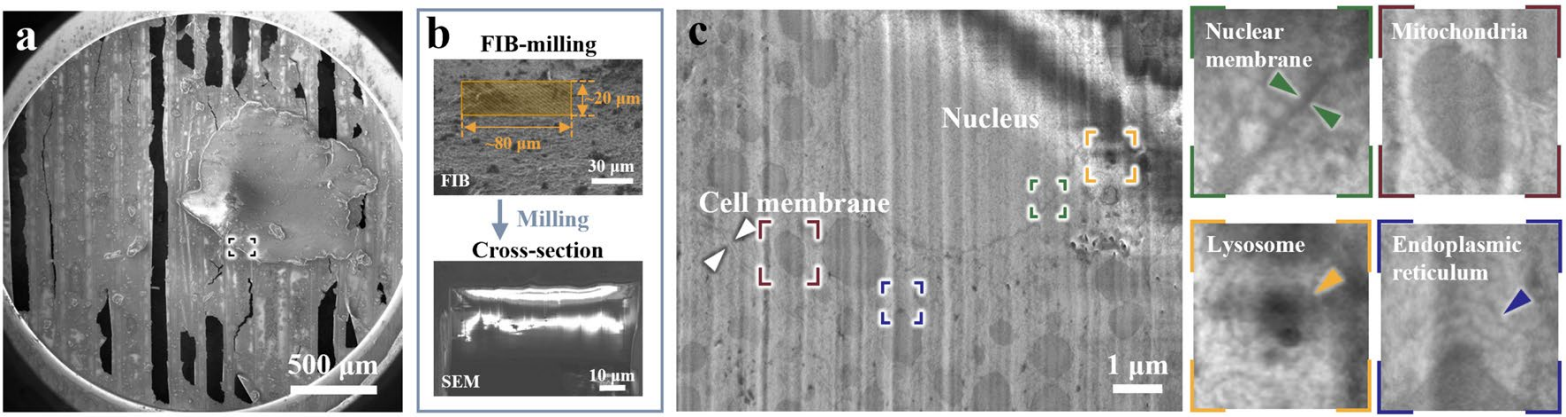
CSEI-based localization on a liver slice. (**a**) Selected region (black box) for CSEI on a liver tissue slice, shown by low-magnification SEM. (**b**) Large window (orange box in the top image, under FIB view) milled for CSEI (bottom image). (**c**) Magnified CSEI area on the surface created by cryoFIB milling of (**b**) showing high contrast features of liver cells. The cell membrane (white arrow) and multiple organelles (colorful boxes and corresponding magnified images shown on the right) in liver cells were observed. All images of CSEI were acquired using Crossbeam 550 (ZEISS Microscopy).

The localization process with CSEI on tissue samples is simple and convenient, achieved by continuously imaging the fresh surface exposed by cryoFIB milling and targeting the objects of interest based on visible cellular features. We used mouse liver tissue as an example to test the localization workflow. First, we randomly selected a region (Fig. 2a) and milled a window of 80 × 20 μm under the cryoFIB view with a shallow beam angle of 18° and a high ion beam current of 5 nA (Fig. 2b). On the generated surface, the ultrastructure of liver cells, such as membranes and various organelles, was clearly visible (Fig. 2c). Such imaging can be performed throughout the milling process.

### Coarse milling to rapidly remove large volumes

The ion beam mills the sample along a small glancing angle nearly parallel to the sample surface; therefore, the volume to be removed increases significantly with increasing sample thickness. We considered two strategies to accelerate the milling: removing the volume around the region of interest with an ion beam along a high incident angle and using high ion beam currents when possible. The bulky sample that remains around the final lamella forms a wall tens of microns high; this poses a problem for thick samples as it often blocks the electron beam during cryoET data collection.

**Figure 3:**
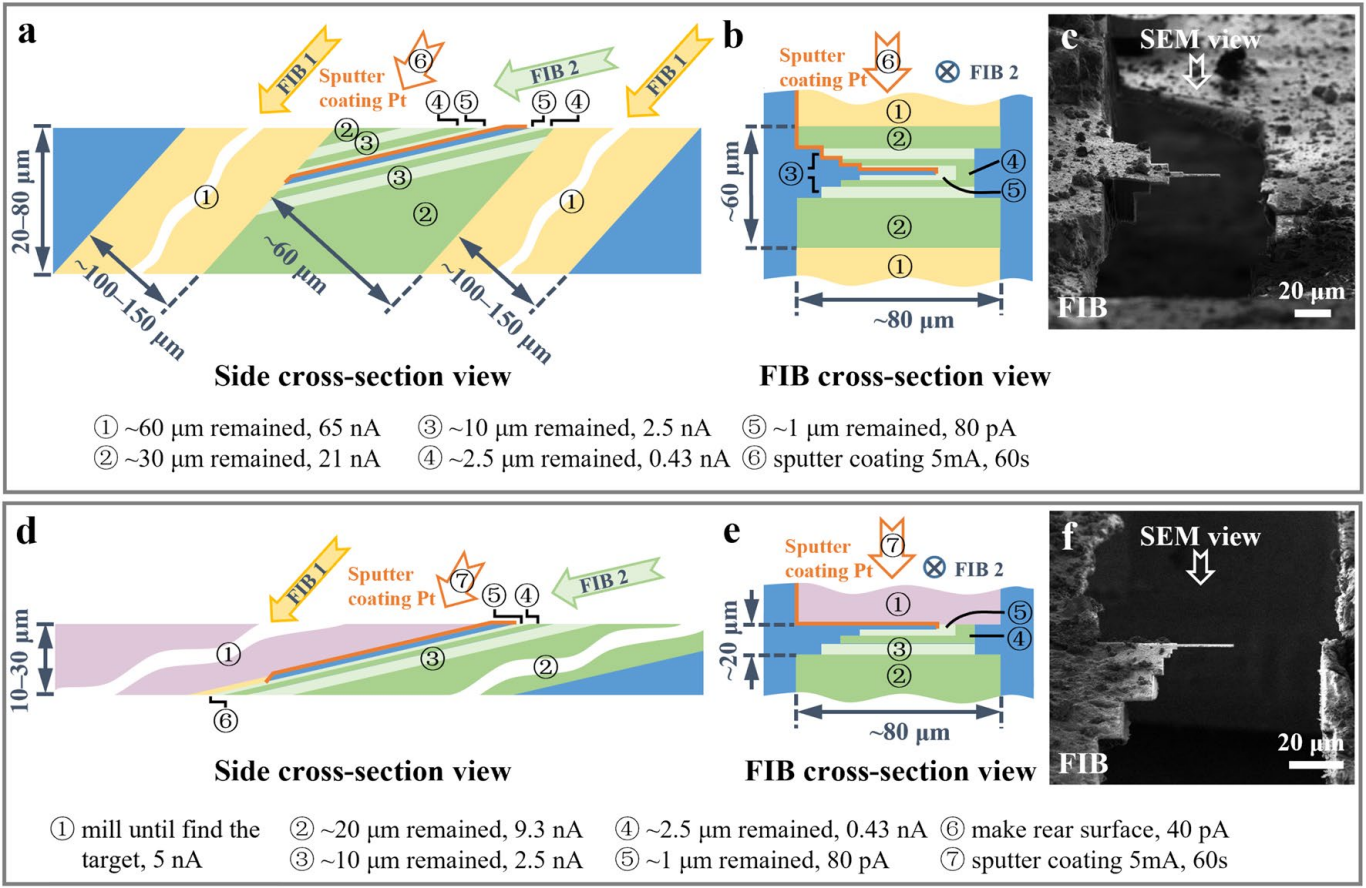
Coarse milling procedure. (**a** and **b**) Schematic diagrams of a typical coarse milling procedure viewed from the side (**a**) and FIB cross-section view (**b**). The yellow and green (light and dark green) strips represent the first and the second sub-steps, respectively, with milling along different ion beam directions (arrows labeled with FIB 1 and FIB 2). (**c**) Image of a liver sample under FIB view after the coarse milling presented in (**a** and **b**). (**d**) and (**e**), Schematic diagrams of a typical coarse milling procedure skipping the first sub-step to avoid damage on the lamella surface for initial CSEI-based localization. The purple strips represent the volumes removed for CSEI-based localization along the ion beam direction of FIB 2. (**f**) Image of a liver sample under FIB view after the coarse milling presented in (**d** and **e**). The strips with different colors and numbers in circles indicate different milling steps and milling parameters (listed at the bottom). The numbers also indicate the sequence of milling and sputter Pt coating (orange edges). The blue volumes and strips are the remaining materials and the final lamella. The FIB images in (**e** and **f**) were acquired using Helios (FEI Company).

We designed the coarse milling process with two sub-steps (see “Methods” Section). In the first sub-step, we milled the sample at a high incident angle (such as 48°), aiming to remove the volumes at the front and back ends of the region of interest (Fig. 3a); this sub-step effectively shortened the milling depth for the further milling with a low ion beam current. The maximum ion beam current is recommended to maximize the milling speed. In our test (Supplementary Fig. 2), removing the volumes in two windows of 80 × 100 μm (under FIB view) on an ~ 80 μm thick sample took less than 1 h when using a beam current of 65 nA. In the second sub-step, the sample was further milled at a shallow incident angle (such as 18°) using sequentially descending ion beam currents (Fig. 3a,b). We created a stepped edge around the lamella to avoid possible beam blocking (Fig. 3b,c). One side of the lamella was disconnected from the bulky sample (Fig. 3b,c). This was achieved by either completely removing the bulky sample on one side (Supplementary Fig. 3) or milling out a gap of 10 μm between the lamella and the remaining bulky sample (Supplementary Fig. 4). This operation is essential to protect the lamellae from being broken by sample deformation (Supplementary Fig. 5) and avoid crashing between the lamella and the remained bulky sample in the future sample transfer.

CSEI can be used throughout the milling process to determine the milling position^17^. In some cases, CSEI is used in the beginning of the coarse milling to find targets sparsely distributed in a large bulky sample. The surface generated during CSEI localization is free of organometallic Pt layers, hence has a low tolerance for irradiation damage. The ion beam used in the first sub-step is incident along a high angle and very strong. Hence, the first sub-step should be skipped to avoid damage on the surface generated during CSEI localization. The second sub-step of coarse milling should be only applied on the other side of the sample (Fig. 3d–f). In such a case using CSEI, we suggest preparing samples with small initial thickness, typically not thicker than 30 μm, in the pre-sectioning step to avoid long milling times.

The final lamella generated by the coarse milling is typically 1 μm thick. The sputter coating is the last step of the coarse milling process, which deposits a conductive Pt layer on the lamella. This conductive layer is useful for eliminating surface charging during future data collection (discussed later). The coating should cover the front, top, and rear surfaces of the lamella (orange layers in Fig. 3a and Supplementary Fig. 6a). In the case that the rear surface of the lamella cannot be effectively coated (Fig. 3d and Supplementary Fig. 6b), the rear surface of the lamella should be milled out using low beam currents (24–40 pA) at a 48° angle to create a sloped surface that can be effectively coated by sputter coating (Fig. 3d).

### Fine milling to generate large, thin lamellae

**Figure 4:**
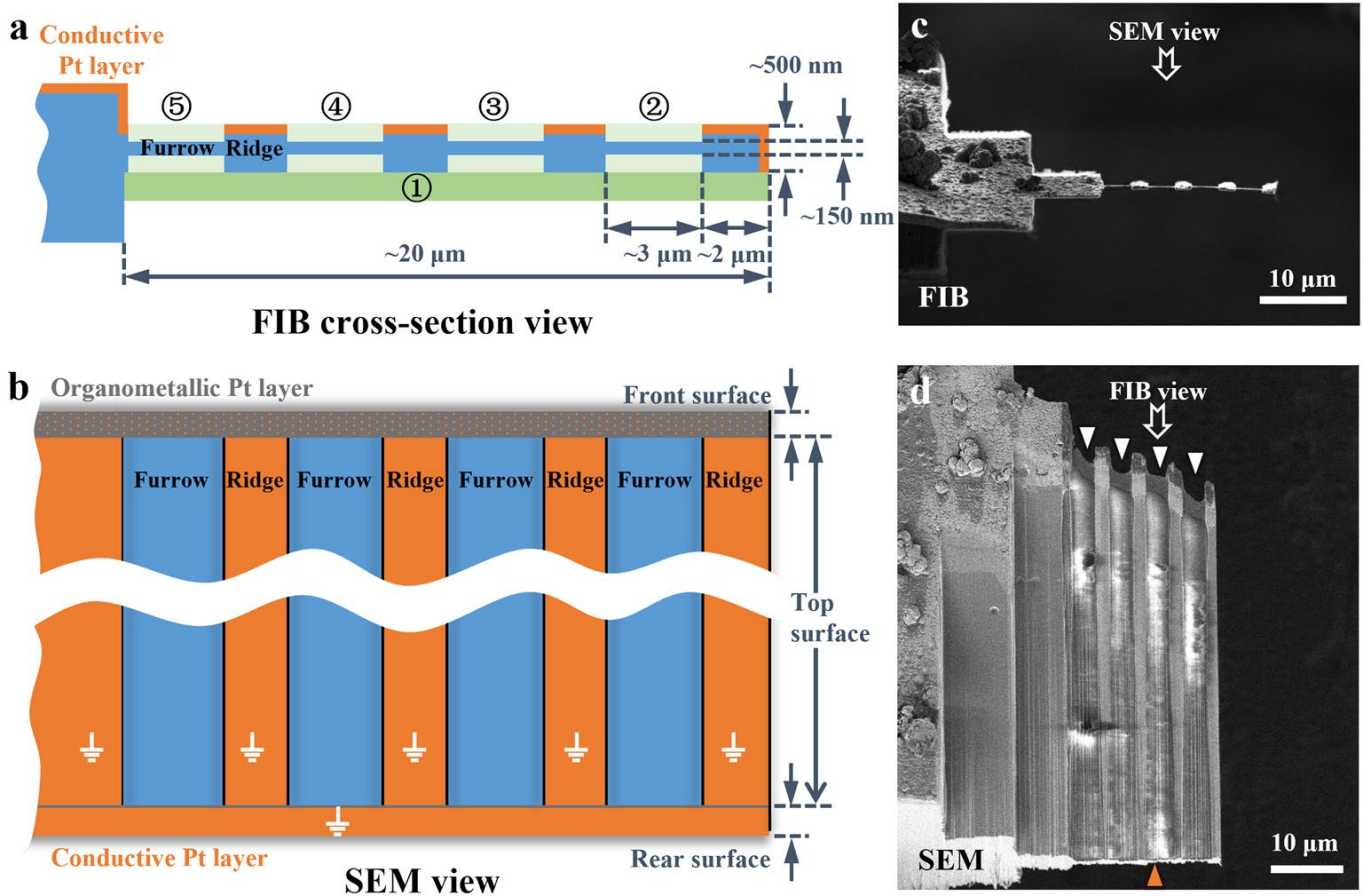
Fine milling procedure and furrow–ridge structure. (**a** and **b**) Schematic diagrams of the fine milling procedure under FIB cross-section view (**a**) and SEM view (**b**). The green strips (dark green and light green) represent the volumes removed in the sequence indicated by the numbers in circles. The orange edges and surfaces indicate the conductive Pt layer. The gray surface is the organometallic Pt layer coated by a gas injection system (GIS). The orange dots on the front surface indicate the conductive Pt layer which might be damaged during fine milling. The front, top, and rear surfaces are defined in Supplementary Fig. 6. The blue volumes and surfaces are the remaining materials. (**c**) and (**d**) Finished lamella of a liver sample created by fine milling under FIB view (**c**) and SEM view (**d**), as presented in (**a**) and (**b**), respectively. The FIB and SEM images in (**c** and **d**) were acquired using Helios (FEI Company).

In the fine milling step, we used a beam current as low as 40 pA to finalize the milling process. The lamellae obtained by coarse milling were usually large, typically 20 μm in width and 40–110 μm in length. The primary obstacle to mill such a large lamella to 100–200 nm thickness is the issue of bending (Supplementary Fig. 7). The bending can be decomposed into lengthwise (along the incident ion beam) bending and widthwise (perpendicular to the incident ion beam and parallel to the lamella plane) bending. The lengthwise bending brings difficulties in measuring the thickness of lamella, hence, terminates the milling before reaching the target thickness. To solve this problem, we designed a furrow–ridge structure to enhance the lamella (Fig. 4), which had been integrated into the milling workflow (see “Methods” Section).

The furrows are the thin areas to be used for future cryoET data collection, and the ridges are the thick ribbons providing mechanical support and charge reduction using the remaining Pt layer coating applied at the end of the coarse milling step (discussed later). We usually set the furrow to ~ 3 µm in width to match the electron beam size during cryoET data collection, and the ridge to ~ 2 µm in width. Accordingly, a lamella with a width of 20 µm can accommodate 4 furrow–ridge pairs (Fig. 4). This furrow–ridge structure can significantly improve the mechanical strength of the large lamella in the lengthwise direction, and, consequently, the success rate of milling lamellae to a thickness less than 150 nm.

Excessively heavy ridges can also cause the lamella to bend in the widthwise direction. Therefore, before milling the furrow–ridge structure, we usually thinned the thick lamella to ~ 500 nm from the bottom surface to reduce the weight of the future ridges while maintaining the Pt layer on the top surface (Fig. 4a). Then, the furrows were milled successively from the disconnected end to the fixed end of the lamella. While the ridges increase the mass of lamella, most lamellae don’t show obvious widthwise bending issues in our experiments. In some situations, previously milled furrows bend when milling successive furrows; milling in a correct order improved the likelihood that each furrow could be milled to the desired thickness before bending. Fortunately, the widthwise bending does not obviously influence cryoET data collection and tomogram reconstruction (Supplementary Fig. 4e–h).

### Using Pt-coated ridges to reduce beam-induced charging

CryoFIB milling of a thick tissue sample exposes a large, hydrated surface that is electrically poor- or non-conductive. Therefore, surface charging was observed on the sample, represented as an electron footprint and image distortion built up around the illuminated area (Supplementary Fig. 8a–g and Supplementary Movie 1). Furthermore, the beam-induced motion was also observed, which increased rapidly as getting closer to the disconnected end (Supplementary Fig. 8 h–i). The motion decreases the quality of images and the accuracy of tracking, and even causes the failure of the data collection near the disconnected end. To solve these problems, we performed additional sputter Pt coating applied at the end of the coarse milling step, which covered the ridges with an electrically conductive Pt layer. The conductive Pt layer reduces the non-conductive surface as much as possible and provides a well-grounded metal layer (on the ridges) tightly surrounding the area for cryoET data collection (the furrows). The electrically conductive region has been shown to be essential for neutralizing the surface charge by making the electron beam touch the conductive area during data collection^24,25^.

**Figure 5:**
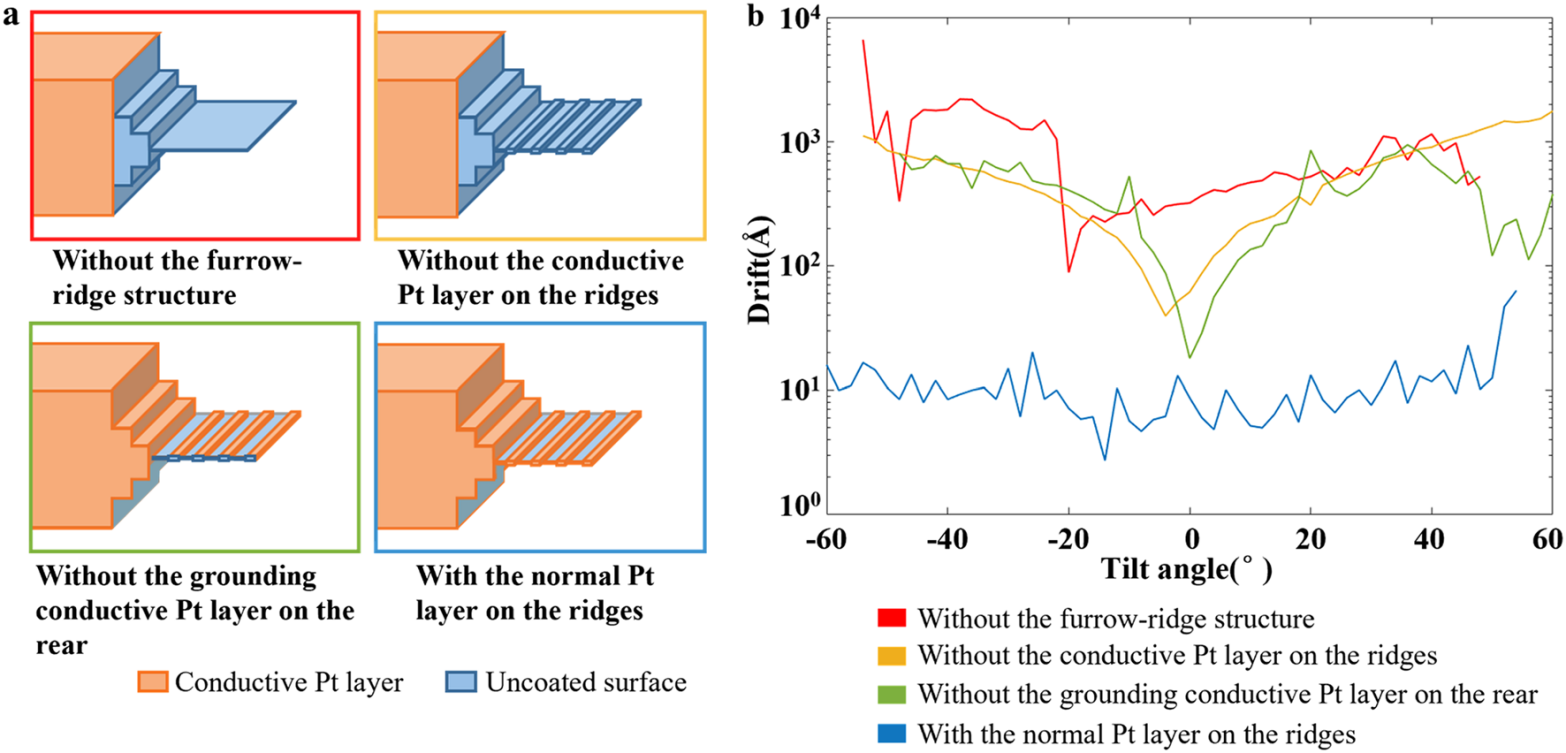
Beam-induced motion near the disconnected end of the lamellae with or without charge-reducing design. (**a**) Schematic diagrams of the four lamellae with different coverage of conductive Pt layers. (**b**) Representative curves (see full data in Supplementary Figs. 8 and 9) of the measured drift (motion) against the tilt angle of cryoET data collection of the four lamellae in (**a**). Different colors of curves and figure boxes indicate different lamella. The red curve is measured at position #1 in Supplementary Fig. 8a, which is 6 μm from the disconnected end. The yellow, green and blue curves are measured at position #1 in Supplementary Fig. 9a,f,k, respectively, which are all in the furrows closest to the disconnected end (~ 3.5 μm from the disconnected end).

Note that the conductive Pt layer on the ridges must be well grounded; this is the reason that the rear surface of the lamella must be sputter Pt coated, as mentioned in the coarse milling step (Fig. 3 and Supplementary Fig. 6). While both the rear and front surfaces of the lamella are Pt coated, the front conductive Pt layer is often damaged by the ion beam, and thus not guaranteed to act as a grounding wire, rendering the rear conductive Pt layer very important (Fig. 4b). To demonstrate the role of the conductive Pt layer on the ridges, we performed three comparisons. First, we prepared a lamella with a furrow–ridge structure but without the conductive Pt layer on the ridges. During cryoET data collection, the furrow close to the disconnected end shows severe motion (Fig. 5 and Supplementary Fig. 9a–e), but the motion is slightly weaker than that of the lamella without a furrow–ridge structure (Fig. 5 and Supplementary Fig. 8). This comparison demonstrates that the mechanical support from the ridge is useful, but not the key factor in reducing motion. Second, we tested a lamella with a furrow–ridge structure on which the conductive Pt layer on the ridge was disconnected from the ground by milling out the rear Pt layer. This lamella exhibits a similar behavior of motion as the previous lamella without the Pt layer on the ridges (Fig. 5 and Supplementary Fig. 9f–j), which confirms that charging is still the problem. Third, we prepared a lamella with a normal furrow–ridge structure, i.e., the ridges had a well-grounded Pt layer. All furrows on the sample show similar and weak motions. Particularly, the motion of the furrow close to the disconnected end is dramatically reduced by approximately 100-folds at high tilt angles (Fig. 5 and Supplementary Fig. 9k–o), demonstrating that charging is the key factor causing severe motion on lamellae with one end disconnected. In the tests above, all the motion curves are U-shaped, indicating that the motion behaved like a swing around the fixed end of the lamellae.

### Example of milling mouse liver tissue

We used mouse liver tissue to demonstrate the complete milling workflow, with the aim of observing collagen fibrils in the liver. Liver fibrosis results in the excessive accumulation of extracellular matrix (ECM). The structure and degree of crosslinking of collagen fibrils, the major ECM structural protein, have important impacts on liver fibrosis recovery^26,27^. Therefore, we expected to observe the ultrastructure of collagen fibrils in mouse liver tissue at the early stage of liver fibrosis. The collagen content has been reported to be low in the early fibrotic liver ECM of the carbon tetrachloride (CCl_4_)-induced mouse model^28^, providing an opportunity to test CSEI in localizing a sparse target.

**Figure 6:**
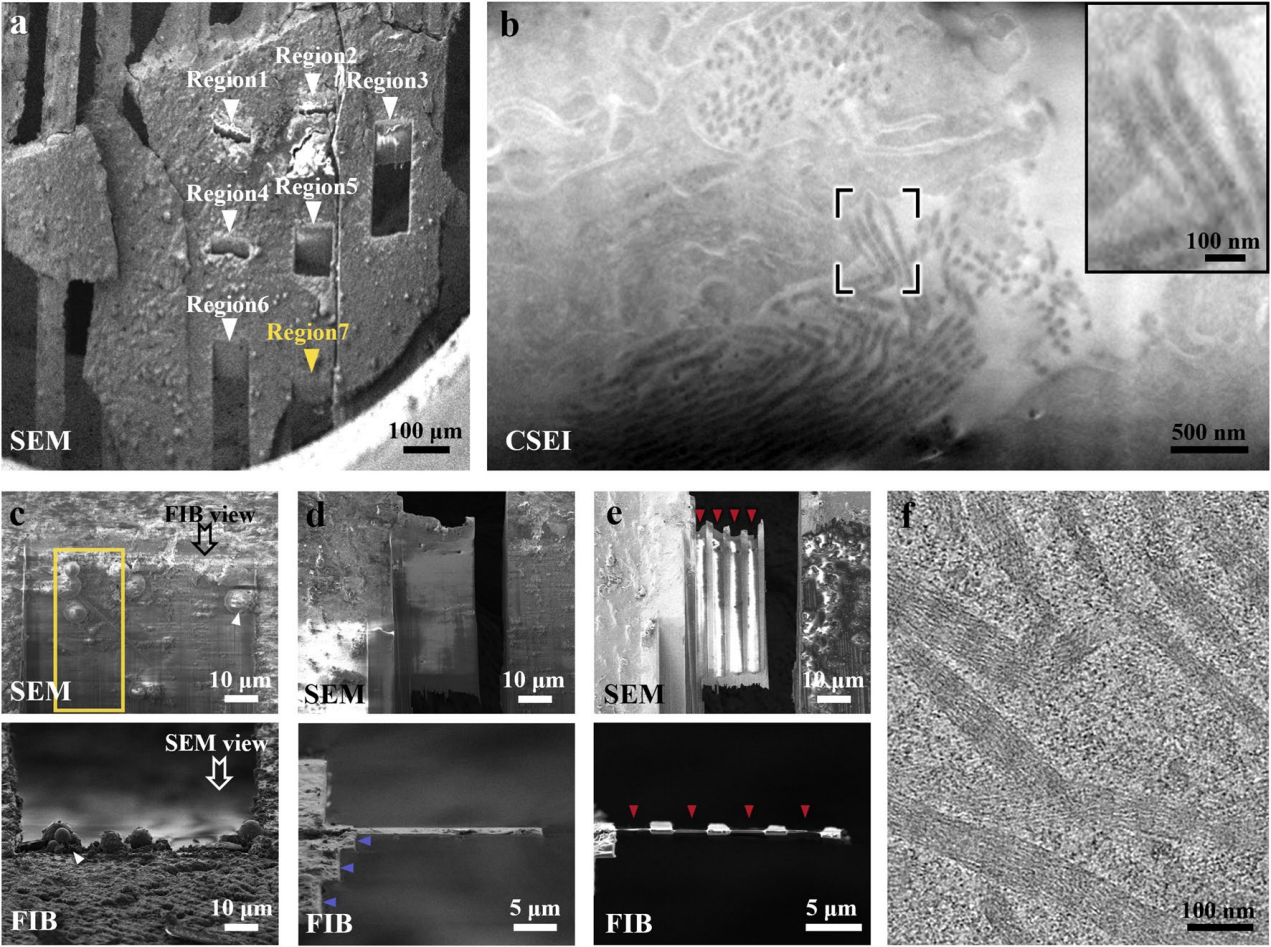
Complete workflow illustrated using a mouse liver sample. (**a**) Pre-sectioned and frozen liver slice under SEM view. Seven regions (arrows) were created for CSEI-based localization. (**b**) Region 7 in (**a**) was viewed by CSEI, showing the target collagen fibrils. The inset image shows magnified fibrils in the black box. (**c**) The top surface of the lamella was milled during CSEI-based localization. The yellow box is the target area. Ice contamination (white arrows) was produced when transferred to another cryoFIB instrument for further milling. (**d**) The liver tissue was coarsely milled to reduce the target area to ~ 1 µm thick. Blue arrows show the stepped edge on one side of the lamella. (**e**) The final lamella after fine milling. Red arrows show the furrows. (**f**) A section-view of a tomogram showing collagen fibrils (Supplementary Movie 2). The imaging method, SEM or FIB, is labeled in the bottom-left of each panel. Crossbeam 550 (ZEISS Microscopy) was used for CSEI-based localization; Helios (FEI Company) was used for further cryoFIB milling procedure.

Following the protocol described above (see “Method” Section and Supplementary Protocol), fresh liver tissue was fixed in glutaraldehyde for 30 min, sliced using a vibratome (Leica VT1200S, Leica Microsystems Company) set to 20 μm, and frozen using HPF. We randomly milled seven regions on the prepared frozen sample (Fig. 6a) and searched for the collagen fibrils using CSEI. Several clusters of collagen fibrils with the characteristic bamboo-like organization were observed in one region (Fig. 6b).

Subsequently, around the collagen fibrils, we generated large lamellae by coarse milling (Fig. 6c–d and Supplementary Fig. 4a–d), and further milled them into thin lamellae with four furrow–ridge pairs using the fine milling protocol (Fig. 6e and Supplementary Fig. 4e–f). Using a 300 kV cryo-electron microscope, we clearly observed collagen fibrils widely distributed in the lamella (Supplementary Fig. 4g). We then collected tilt series data and performed tomography reconstruction. The tomograms showed high contrast features of the D-period of collagen fibrils^26,29^, and the lamella thickness was approximately 130 nm (Fig. 6f, Supplementary Fig. 4h, and Supplementary Movie 2). In another sample, we obtained a thinner lamella of ~ 100 nm thick, in which more cellular ultrastructures were observed, including mitochondria and endoplasmic reticulum (Supplementary Fig. 10 and Supplementary Movie 3).

## Discussion

In this study, we proposed a complete workflow for milling large tissue samples using cryoFIB, which involved pre-sectioning, on-the-fly localization, and rapid milling. This workflow required only the basic functions of a cryoFIB instrument, including secondary electron imaging and focused ion beam milling. CSEI provides a convenient way to accurately locate the samples and eliminate reliance on fluorescence labels. More importantly, CSEI is very reliable on various surfaces created by cryoFIB milling, which allows for constant imaging and the determination of the 3D position of target objects during milling. Together with our high-efficiency cryoFIB milling strategy, preparing the thin lamella starting from a large tissue has become more achievable. Finally, we demonstrated the entire workflow using a mouse liver and obtained high-quality tomograms of the collagen fibers sparsely distributed in the liver sample.

Localization using CSEI is applied on newly exposed surfaces continuously milled by FIB. The lateral resolution of CSEI localization depends on the imaging resolution of SEM (typically at the nanometer level), and the axial resolution depends on the minimum step of FIB milling (as high as several nanometers level)^15^. Both the lateral and axial resolutions are much better than those of cryoCLEM. The field of view of CSEI can be as large as ~ 50 µm × 50 µm, which is usually sufficient for widely distributed targets in a large tissue sample. For sparely distributed targets, incorporation with cryoCLEM for initial localization ahead of CSEI should be a good way to extend the view range for localization.

The bottleneck for thinning tissue is the low efficiency. We developed two strategies to solve this problem. The first strategy involved coarse milling using an ion beam with as high as 65 nA current and a high incident angle to rapidly remove the volume surrounding the target object and shorten the milling depth for further milling at a shallow angle. The second strategy was to generate a large lamella with a width and length of tens of micrometers, allowing us to expose a large sample area for cryoET analysis. Combining the two strategies, the milling efficiency starting from a large tissue sample was on par with that starting from a thin single-cell sample. Compared with other published methods (Supplementary Table 4), the milling efficiency of our method is higher than those using the cryo-lift out^9,30^ and the waffle method^31,32^. As for the VHUT-cryoFIB method^8^ requiring additional cryo-ultramicrotomy operations, our method is much more efficient when counting the total time of sample preparation. Importantly, different from other published methods^8,9,30–32^, our method is just based on the fundamental functions and operations of the cryoFIB and HPF instruments, and doesn’t need customized instruments and additional tools (Supplementary Table 4). The major problem currently affecting the success rate is ice contamination, which occurs during the transfer process after milling and can significantly reduce the area suitable for cryoET data collection. Efforts to reduce ice contamination are still necessary.

The furrow–ridge structure is the key to obtaining high-quality cryoET data. This structure allows us to successfully obtain high-quality lamellae as thin as 100 nm. Small lamella thickness is essential for achieving sub-tomogram averaging at near-atomic resolution. Furthermore, the well-grounded conductive Pt layer on the ridge provided an effective way to relieve the beam-induced motion disturbing cryoET data collection and subsequent analysis. Compared with the whole-lamella post-milling Pt sputtering^33^, the areas for data collection (i.e. the furrows) in our method are free of Pt layers which may introduce additional noises in the following data collection. And the probable damage from the coating process is also avoided, since the sample layer with Pt coating has already been removed during the final fine milling. Importantly, benefiting from the charge-reducing design, the reduced motion improved the accuracy of tracking and the quality of micrographs, accordingly, the accuracy of tomography alignment.

Widthwise bending has been observed in some lamella with the furrow–ridge structure in our experiments. When using an adjacent furrow for focusing in cryoET data collection (see Supplementary Protocol H2), the widthwise bending can cause a defocus offset since the areas for exposure and focusing have different heights. Because the bending is minor, the influence of the defocus offset is often negligible. Besides the defocus offset issue, the widthwise bending didn’t have remarkable influences in the following cryoET processes in all our experiments (Supplementary Fig. 4h and Supplementary Movie 2). The widthwise bending is due to the lack of support on the disconnected end of the lamella. The waffle method^31,32^ used a notch design to provide support on the disconnected end. However, the distance between the disconnected end with a notch and the remained bulky sample is ~ 200 nm (~ 10 μm in the present design without the notch), and, hence, the notch design cannot survive severe deformation as large as several micrometers (Supplementary Fig. 5a–b).

The width of ridges should be optimized for different experimental conditions. Since the ion beam gets more and more divergent in the far end, the ridges often become narrower on the rear of the lamella than that on the front part (Fig. 4d). If the width of the rear ridges is too narrow, the conductive Pt layer on the rear ridges may be disconnected from the conductive Pt layer used for grounding on the rear surface, resulting in the failure of the charge-reducing design. A suitable width of ridges must be tested and set on different instruments or ion beam conditions.

In summary, our workflow for tissue sample preparation eliminates the major obstacles in the preparation of large tissue samples for cryoET and efficiently broadens the applicable range of cryoET to nearly any large biological sample.

## Methods

### CCl_4_-intoxicated liver fibrosis mouse model

Adult C57BL/6 mice (8 weeks, male) were purchased from Cyagen Biosciences (Suzhou, China) and acclimatized in cages for 3 days. The liver fibrosis mouse model was established by intraperitoneally injecting 12.5% of CCl_4_ (Innochem, China) in mineral oil (1/7, v/v) at a dose of 0.01 ml/g body weight twice a week for up to 8 weeks (for early fibrosis). Healthy mice only received isovolumic mineral oil in the same manner and were used as controls. All mice were housed and bred at 23 ± 2 °C, under a 12-h light–dark cycle with standard chow and water ad libitum. Mouse studies were approved by the Ethics Committee of Beijing Friendship Hospital, Capital Medical University, and carried out in accordance with ARRIVE guidelines (https://arriveguidelines.org). All methods were carried out in accordance with relevant guidelines and regulations.

### Liver tissue sample acquisition and vibratome sectioning

Mice were euthanized using excess pentobarbital sodium. Liver tissues were dissected and cut into small cuboids (with an edge length of 1–3 mm) with a razor blade. Subsequently, the liver tissue cuboids were washed 2–3 times in cold phosphate buttered saline (PBS) and submerged in 2.5% glutaraldehyde (Electron Microscopy Sciences) for 30 min at room temperature. Subsequently, liver tissue cuboids were washed 2–3 times in cold PBS and submerged in 4% liquid agarose (AGARSE II, 0815-5G, Amresco) at approximately 40 °C. After agarose solidification, the agarose was mounted on the sample stage of a vibratome (Leica VT1200S, Leica Microsystems). The buffer tray of the vibratome was filled with cold PBS. The vibratome was set to section at a thickness of 10–50 µm (sectioning frequency: 85 Hz; amplitude: 1 mm; sectioning speed: 0.5 mm/s). The liver slices were then carefully transferred to cold PBS using a brush.

### High-pressure freezing of liver tissue

The liver slices (immersed in PBS) were picked up using a Perfect Loop (Diatome, DZ8) and loaded onto glow-discharged grids coated with lacey carbon film (parallel bars, Cu, 150 mesh, Zhongjingkeyi Technology, AG150P) (Fig. 1b). Next, grids with tissue slices were blotted on filter paper to remove excess liquid (~ 5 s) and transferred into aluminum carriers (6 mm aluminum specimen carrier Type A, 200 µm recess, Leica Microsystems), which were previously filled with 2-methylpentane (Sigma, M65807). The aluminum carriers were then covered with sapphire discs and immediately transferred to a high-pressure freezer (Leica HPM100, Leica Microsystems). After cryo-freezing, the aluminum carriers were transferred into a cryo-ultramicrotome chamber (Leica EM UC7 + FC7, Leica Microsystems) at − 150 °C to melt the frozen 2-methylpentane (melting point, − 154 °C). Thereafter, grids were separated from the aluminum carriers and placed in the cryo-ultramicrotome chamber for ~ 20 min to remove excess 2-methylpentane. Finally, grids were transferred to grid boxes and stored in liquid nitrogen.

### Preparation for cryoFIB milling

The frozen grids containing tissue slices were mounted into Autogrids (Thermo Fisher Scientific). The Autogrids were then mounted onto a sample shuttle. Note that the direction of the Autogrid should be aligned such that the grid bars point toward the incident direction of the ion beam (see Supplementary Protocol D1-5).

The shuttle was transferred to the prep-stage in the prep chamber of the cryo-transfer system (PP3010T, Quorum Technologies), which was pre-cooled to − 180 °C. The sample was sublimated under a high vacuum in the prep chamber at − 150 °C for 30–90 min to ensure complete removal of 2-methylpentane and exposure of the tissue bulk. After sublimation, the temperature of the prep-stage was set back to − 180 °C. The shuttle was then transferred to the cryo-stage in the SEM chamber (Helios Nanolab G3 UC, FEI company) and elevated to a position such that the grid was 8 mm from the SEM tip. The acceleration voltage and beam current of the SEM were set to 2 kV and 0.4 nA, respectively. The organometallic Pt gas in the gas injection system (GIS) was preheated to 42 °C. Then, electron-beam-induced organometallic Pt deposition was performed for 40 s with a continuous electron beam scanning over the entire coated area at a magnification of 100× . To improve the conductivity, the shuttle was transferred back to the prep chamber for sputter coating (5 mA, 60 s) to cover the organometallic Pt layer with a thin conductive Pt layer.

### CSEI-based localization

The Autogrids were mounted onto a ZEISS-customized shuttle and transferred to a Crossbeam 550 FIB-SEM (ZEISS Microscopy, Oberkochen, Germany) using a QUORUM PP3010Z transfer system. Throughout the procedure, the samples were kept below − 170 °C. The cryo-stage was tilted to 8° to produce lamellae at a shallow angle (18° relative to the grid plane). A section surface of 80 μm in width was milled by cryoFIB with the following settings: 30 kV acceleration voltage, 5–7 nA ion beam currents. The cryoFIB milling was continuously performed on the side facing to the electron beam. The newly exposed surface was simultaneously imaged by CSEI using the in-lens and in-chamber detectors at 3 kV acceleration voltage. The SEM imaging settings included an electron beam current of 50 pA, image size of 1024 × 768 pixels, dwell time of 1.8 μs (scan speed of 5), and repetitive scans of 20 times. The in-lens/in-chamber mixed detection was performed using the smartSEM (ZEISS Microscopy) with a mixing ratio between 0.5 and 0.7. The FIB milling was stopped immediately once the target was found. Further milling should be performed only on the other side.

### CryoFIB milling procedure

A dual-beam FIB/SEM (Helios Nanolab G3 UC, FEI Company) with a cryo transfer system (PP3010T, Quorum Technologies) was used for lamella preparation. The angle between the electron beam (for SEM) and ion beam (for cryoFIB) was 52°. A 2 kV accelerating voltage and a 50 pA electron beam current were used for SEM imaging, and a 30 kV accelerating voltage and a 40 pA ion beam current were used for cryoFIB imaging. Throughout the cryoFIB milling process, the samples were kept below − 170 °C and the system vacuum pressure was approximately 1.5 × 10^−4^ mbar.

The entire cryoFIB milling procedure consisted of two steps: coarse milling and fine milling (see Supplementary Tables 1 and 2 for typical parameters). The coarse milling procedure was performed in two sub-steps (see Supplementary protocol for additional guidance). In the first sub-step, the sample stage was tilted such that the incident direction of the ion beam and the grid plane formed a high angle of 48°. CryoFIB milling using an ion beam current of 65 nA was performed to remove sample volume in two rectangular windows (80 × 100 μm under FIB view) using the Rectangle Pattern (named in the FIB user interface) at the front and back ends of the target position, resulting in a slab of 60 μm in thickness under FIB view (Supplementary Figs. 2 and 3a–b). In the second sub-step, the FIB incident angle was adjusted to the final milling angle of the lamella (between 13–18°) (Supplementary Fig. 3d). The second sub-step of the coarse milling process was performed with stepwise reduction of the ion beam current (from 21 nA, 9.3 nA, 2.5 nA, 790 pA, to 80 pA) and the width of rectangle window (from 70 μm, 50 μm, 30 μm, 25 μm, to 20 μm) to create a stepped edge around the lamella. The lamella was thinned to approximately 1 μm in thickness by stepwise movement of the rectangular windows closer to the final lamella (Supplementary Fig. 3e–g). For the lamellae previously located by CSEI**,** only the second sub-step of the coarse milling process was performed on the bottom surface using stepwise reducing ion beam current (from 9.3 nA, 2.5 nA, 790 pA to 80 pA) (Supplementary Fig. 4). The width of the rectangular milling window was gradually reduced to create a stepped edge on one side of the lamella. The rear of the lamella was milled out using a 40 pA ion beam current at a 48° angle. In addition, one side of the lamellae was disconnected from the bulky sample during coarse milling. Lamellae were transferred to the prep chamber for sputter coating (5 mA, 60 s). Fine milling was performed using a 40 pA ion beam current to prepare the final lamellae with 4 furrow–ridge pairs. The furrows and ridges had widths of 3 μm and 2 μm, respectively (Supplementary Fig. 3i).

### CryoEM imaging and cryoET data collection

After cryoFIB, the Autogrids were loaded into a Krios Cassette, ensuring that the milling direction of lamellae was perpendicular to the tilt axis for cryoET data collection. The lamellae were imaged using an Titan Krios microscope (FEI Company) operated at a voltage of 300 kV and equipped with a Quantum post-column energy filter (Gatan) and a K3 Summit direct electron detector (Gatan). Image acquisition was controlled using SerialEM^34^. Montages of the whole lamella were acquired at 2250×magnification. Two magnifications were used for cryoET data collection. The collagen fibril tomograms (Fig. 5f and Supplementary Fig. 5h) were acquired at a magnification of 33,000× (pixel size of 2.12 Å). The tomogram shown in Supplementary Fig. 10a was acquired at a magnification of 19,500 × (pixel size of 3.65 Å). Tilt series were acquired using a bidirectional tilt scheme starting from − 20° relative to the lamella plane (i.e. from − 7° to 67°, then from − 9° to − 47° for a 13°-tilted lamella, and from − 2° to 68°, then from − 4° to − 46° for an 18°-tilted lamella) with a tilt angular step of 2°. The target defocus was in the range of − 4 to − 6 μm. Micrographs with 8 frames were recorded with a 0.075 s per frame exposure time at each tilt angle, resulting in an electron dose of 2 e^−^/Å^2^. The total accumulated electron dose for a tilt series is ~ 116 e^−^/Å^2^. During cryoET data collection, the electron beams for recording/tracking/focusing illumination were controlled to ensure the touch with the ridges to eliminate charging. The tracking/focusing area was selected in an adjacent furrow close to the recording area.

### Image processing

Dose fractionated images recorded by the Gatan K3 Summit camera were motion-corrected using MotionCorr2^35^. All tilt series were aligned using patch tracking and reconstructed by Simultaneous Iterative Reconstruction Technique (SIRT) using the IMOD software package (version 4.9.12)^36^. The contrast of the tomogram was enhanced by denoising and missing-wedge correction using IsoNet^37^ with default settings. Tomogram segmentation and 3D visualization were performed using Amira 20.2 (Thermo Fisher Scientific, Mercury Computer Systems).

### Motion analysis

Different types of lamellae were prepared using a dual-beam FIB/SEM (Helios Nanolab G3 UC, FEI Company). The first type was a lamella without the furrow–ridge structure. The lamella was milled to ~ 150 nm thickness. The second type was a lamella with a furrow–ridge structure but without the conductive Pt layer on the ridges. The lamella contained 4 furrow–ridge pairs, but no additional sputter Pt coating was applied following coarse milling. The third type was a lamella with a furrow–ridge structure, but the conductive Pt layer on the ridge was disconnected to the ground by milling out the rear Pt layer at a 48° milling angle after sputter coating.

The above lamellae and a lamella with a normal furrow–ridge structure were transferred to the Titan Krios electron microscope (FEI Company). Tilt series were collected at a magnification of 33,000× with a pixel size of 2.12 Å. Other settings are the same as those of cryoET data collection mentioned above. Some micrographs at high tilt angles were unable to be collected due to the failure of tracking caused by severe motion, resulting in a varying number of micrographs in a tilt series. MotionCor2^35^ was used for whole-frame motion correction. Drift at each tilt angle was measured by calculating and summing the displacement between adjacent frames of the 8-frame micrograph.

## Supplementary Information

Supplementary Information 1.

Supplementary Video 1.

Supplementary Video 2.

Supplementary Video 3.

Supplementary Information 2.

## Data Availability

The tomograms of collagen fibrils have been deposited into the Electron Microscope Data Bank (EMDB) with the accession code EMD-33910 for Fig. 5f and the accession code EMD-33911 for Supplementary Fig. 4h. The tomograms of mice liver tissue are shown in Supplementary Fig. 10a has been deposited into EMDB with the accession code EMD-33912, and the corresponding one processed by IsoNet was deposited with the accession code EMD-33913. Also the datasets used and/or analyzed during the current study are available from the corresponding author on reasonable request.
