# Supplementary material for: CryoFIB milling large tissue samples for cryo-electron tomography
Source: Sci Rep. 2023 Apr 11;13:5879. doi: 10.1038/s41598-023-32716-z (PMC10090186; doi:10.1038/s41598-023-32716-z)
Supplement: Supplementary file 5 — Supplementary Information 2. [file 41598_2023_32716_MOESM5_ESM.doc]

**Supplementary Figures and Legends**


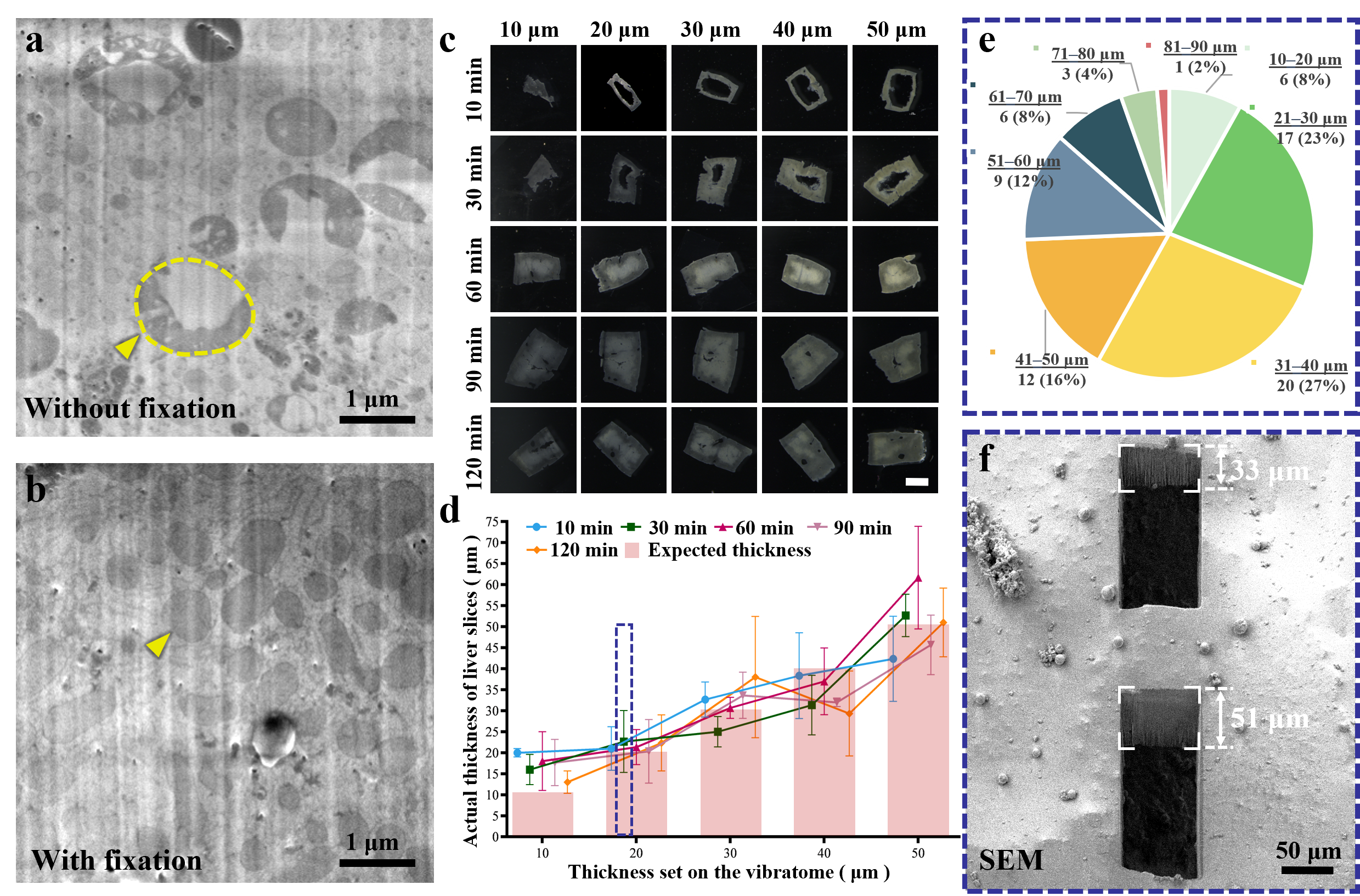


**Supplementary Figure 1. Chemical fixation and pre-sectioning of liver tissue. a** and **b,** CSEI of a frozen hydrated liver tissue sample processed without (**a**) or with (**b**) fixation ahead of pre-sectioning. The pre-sectioning process required approximately 15 min and caused some cell damage. The cellular contents released from broken cells may have influenced adjacent liver cells in the unfixed sample, resulting in mitochondria with a tumid feature (yellow arrow and dashed circle in **a**). 2.5% glutaraldehyde fixation for 30 min reduced this effect, as indicated by the compact mitochondria shape (yellow arrow in **b**). **c,** Photos of liver tissue slices prepared with different pre-sectioning thicknesses (labeled at the top) and fixation times (labeled on the left) using 2.5% glutaraldehyde. The liver tissues fixed for 10 min exhibited a large broken central region. The size of the broken region was reduced with increasing fixation time. Liver slices fixed for 60–120 min were intact. The brightness of the central region also gradually darkened with increasing fixation time, which may indicate more glutaraldehyde diffusion into the central region. **d.** Statistical analysis of the actual thickness of liver slices prepared using a vibratome. The expected thickness is the thickness set on the vibratome, shown as pink bars. Each data point was measured from at least three samples and shown as the mean and standard deviation (error bars). The large error bars indicate a large variation between the thickness set on the vibratome and the actual slice thickness. Data points measured on samples with different fixation times are shown with different colors. The samples from one of the conditions (dark-blue dotted box) are individually analyzed in (**e** and **f**). **e,** Statistical distribution of the sample thickness measured from the 74 samples included in the condition labeled by the blue dotted box in (**d**). **f,** Two windows penetrating the sample were milled by FIB along an incident angle of 48^o^ relative to the sample surface. Under SEM view, the walls of the two windows exhibited differing heights of 33 μm and 51 μm, respectively, demonstrating the uneven thickness of the liver slices prepared by the vibratome.


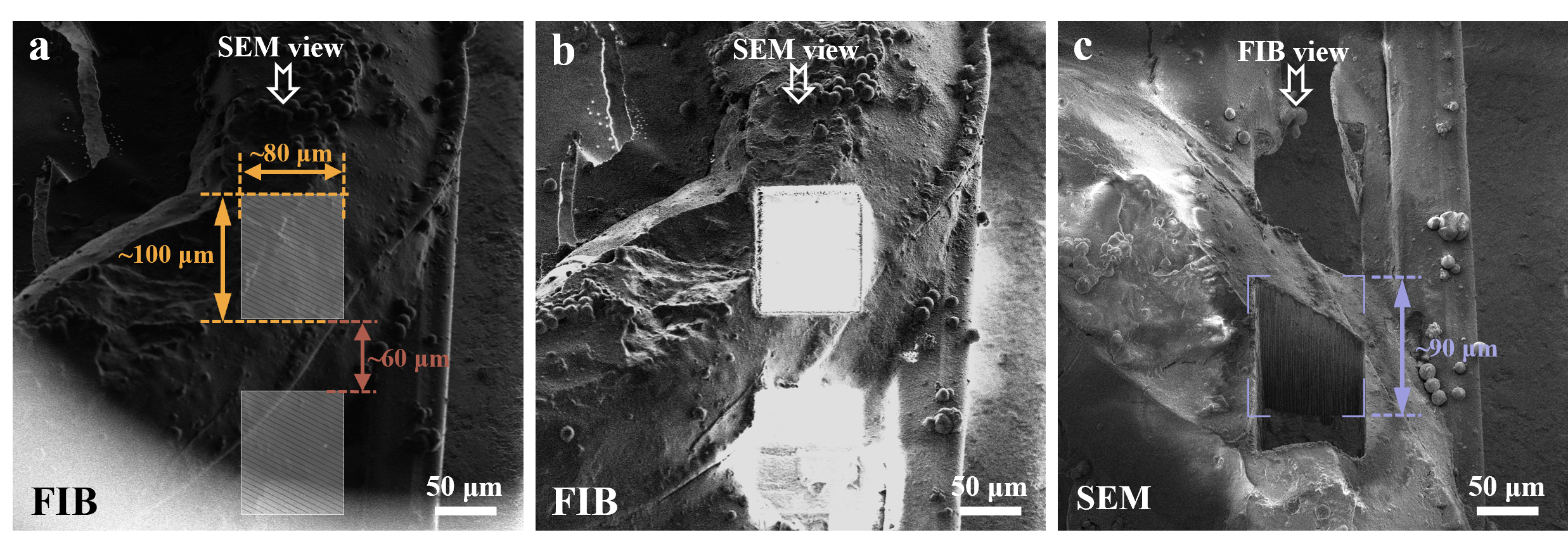


**Supplementary Figure 2. Example of the first sub-step of coarse milling. a**, Two windows of ~80×100 µm^2^ separated by 60 µm were planned for milling under FIB view. The ion beam was along a large incident angle of 48° relative to the grid plane. **b**, The two windows were milled using a 65 nA ion beam current, imaged under FIB view. **c**, The SEM view of the two milled windows. The walls of the window were ~90 μm in height under SEM view. Considering the angle between the electron beam (for SEM) and the ion beam (for cryoFIB) was 52°, the actual thickness of the sample was ~84 μm. All images were acquired using Helios (FEI Company).


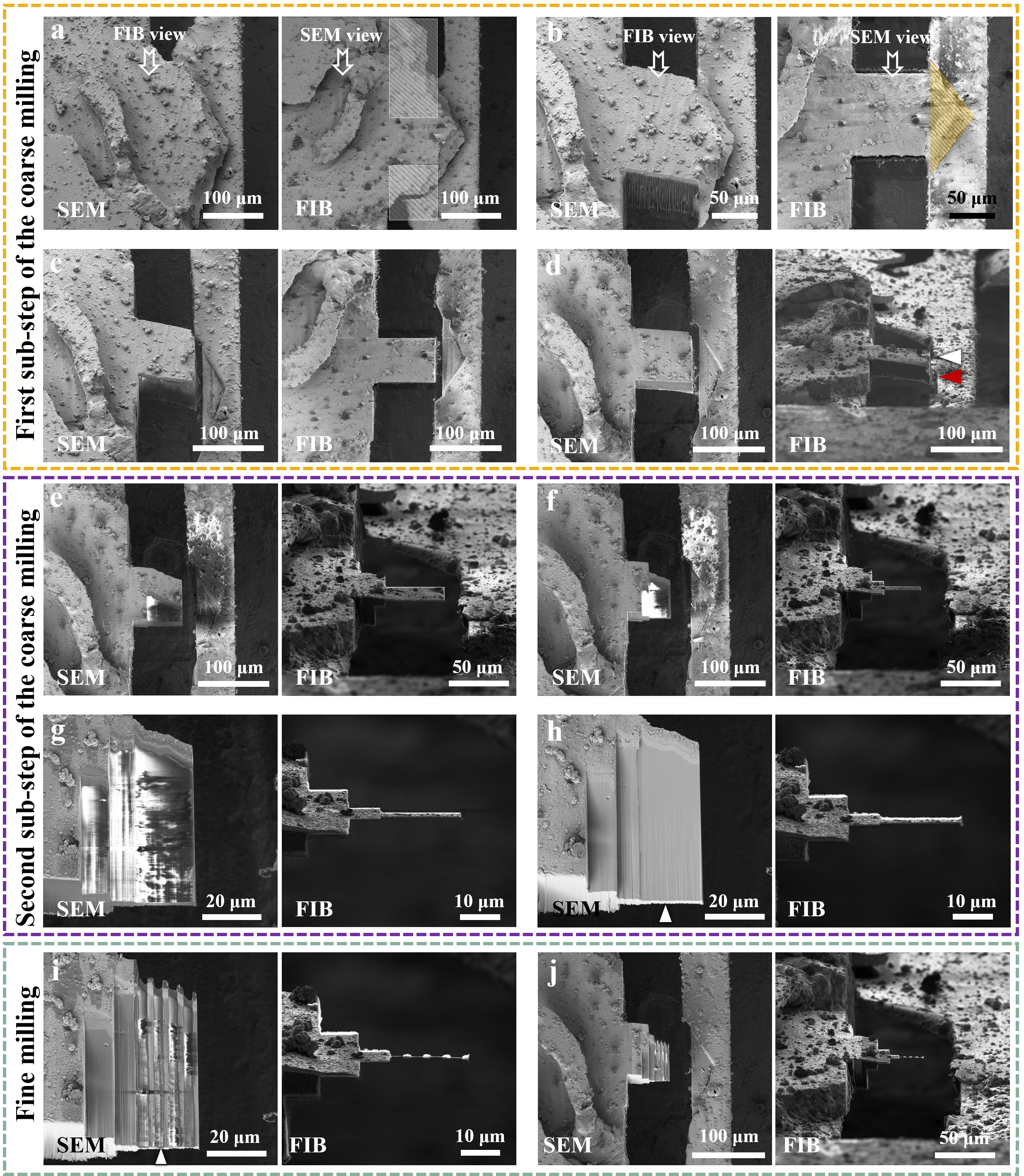


**Supplementary Figure 3. Example of the complete milling procedure.** A frozen hydrated mouse liver sample was used for this example. **a,** The sample was tilted such that the ion beam milled along an incident angle of 48°. Two windows were planned under the FIB view for the first sub-step of coarse milling. A pair of images under SEM view (left) and FIB view (right) are shown for all panels. **b**, The two windows were milled using a 65 nA ion beam current at an incident ion beam angle of 48°, as specified in (**a**). **c,** An edge of the sample (yellow triangle area in **b**) was removed using a 65 nA ion beam current at an incident ion beam angle of 48°. The edge was loosely attached to the grid bar and may have been unstable once the target area was thinned; therefore, removing the edge ahead of further milling may have helped to maintain the stability of the target area. **d,** The sample viewed after adjusting the incident ion beam angle to 13° relative to the grid plane. This angle was maintained in subsequent milling. The target milling position should be chosen on the original sample surface covered by an organometallic Pt layer (white arrow) but not the freshly milled surface (red arrow). **e,** The sample was milled to 10 μm thick in the second sub-step of coarse milling using a beam current of 2.5 nA. **f**, The sample was milled to 2.5 μm thick in the second sub-step of coarse milling using a beam current of 0.43 nA. **g**, The sample was milled to 1 μm thick in the second sub-step of coarse milling using a beam current of 80 pA. This was the final thickness following coarse milling. **h,** The sample was processed by sputter coating using a 5 mA current for 60 s. The rear surface of the lamella is indicated by a white arrow. **i,** The final lamella with the furrow-ridge structure. The lamella had a width of 20 μm, length of ~50 μm, and thickness of ~130 nm, and contained 4 furrow-ridge pairs. The furrows and ridges have widths of 3 μm and 2 μm, respectively. The bright edge on the rear surface of the lamella (white arrow) was the metallic Pt layer generated during sputter coating. **j,** The overall view of the lamella under low magnification. All images were acquired using Helios (FEI Company).


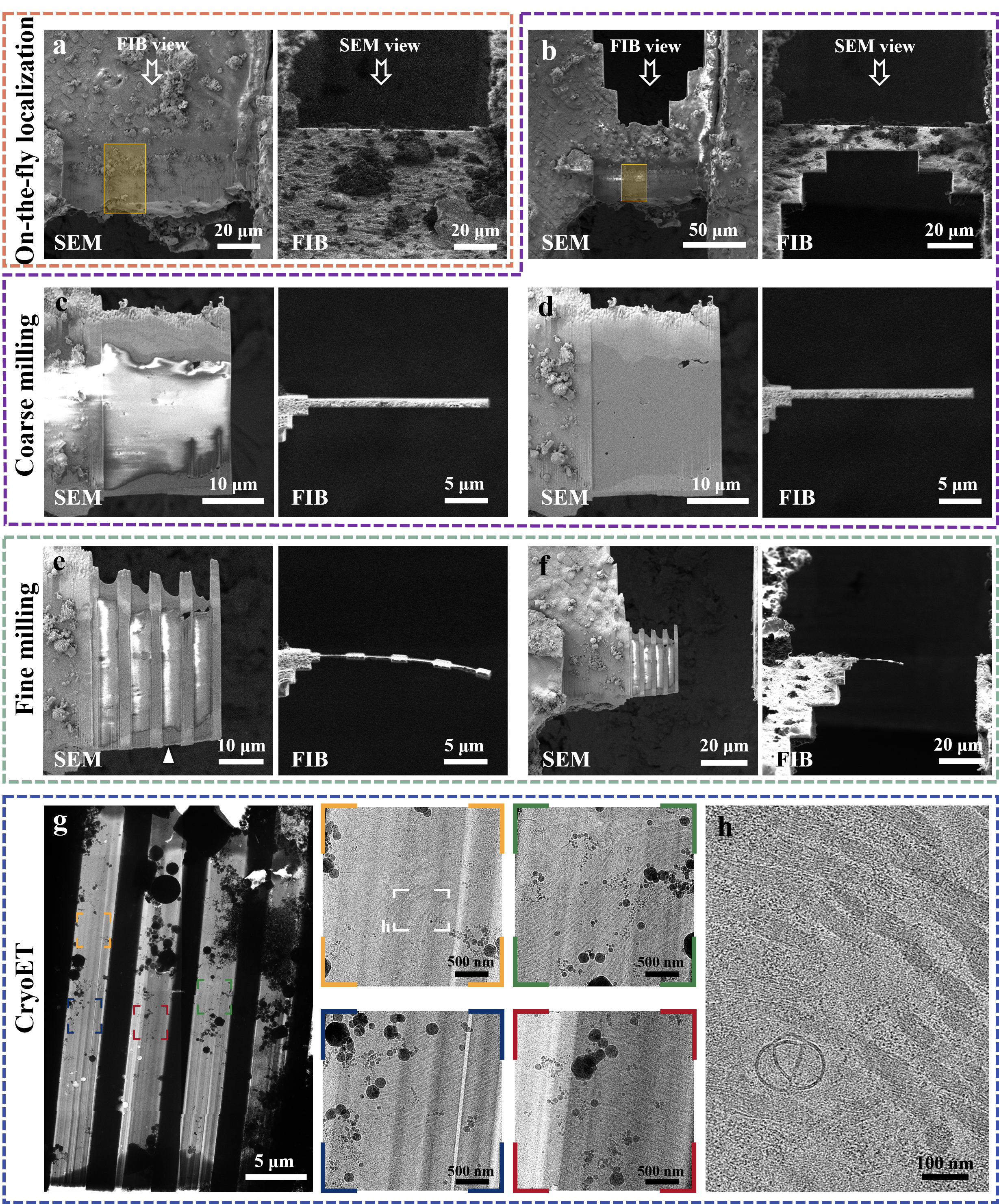


**Supplementary Figure 4. Example of a complete workflow to observe collagen fibrils in mouse liver tissue.** This figure shows the cryoFIB milling procedure on one side of the lamella after the objects of interest, collagen fibrils, were located by CSEI. Note that the first sub-step of coarse milling was skipped in order to avoid radiation damage caused by the strong ion beam. **a,** The surface milled for CSEI localization. The target region containing collagen fibrils is marked by the yellow box. Images under SEM view (left) and FIB view (right) are shown in panels (**a**) through (**f**). **b,** The lamella milled to 10 μm thick during coarse milling. The target region containing collagen fibrils is marked by the yellow box. **c,** The lamella further milled to 1 μm thick, which was the final thickness following coarse milling. Note that the right side of the lamella was disconnected from the bulky sample by a milled gap of 30 μm. **d,** The sample was processed by sputter coating. **e,** The final lamella with 130 nm thickness, generated after fine milling. The edge on the rear surface of the lamella (white arrow) was the metallic Pt layer generated during sputter coating. **f,** The overall view of the lamella under low magnification. **g,** CryoEM image of the lamella under low magnification. The collagen fibrils (colorful boxes and corresponding magnified images shown on the right) in different areas of the lamella were observed. **h,** A sliced view of a tomogram collected from the labeled region indicated by the white box in (**g**), which shows characteristic features of the collagen fibrils (**Supplementary Movie 2**). Zeiss Crossbeam 550 (ZEISS Microscopy) was first used for CSEI-based localization, and then Helios (FEI Company) was used for further cryoFIB milling.


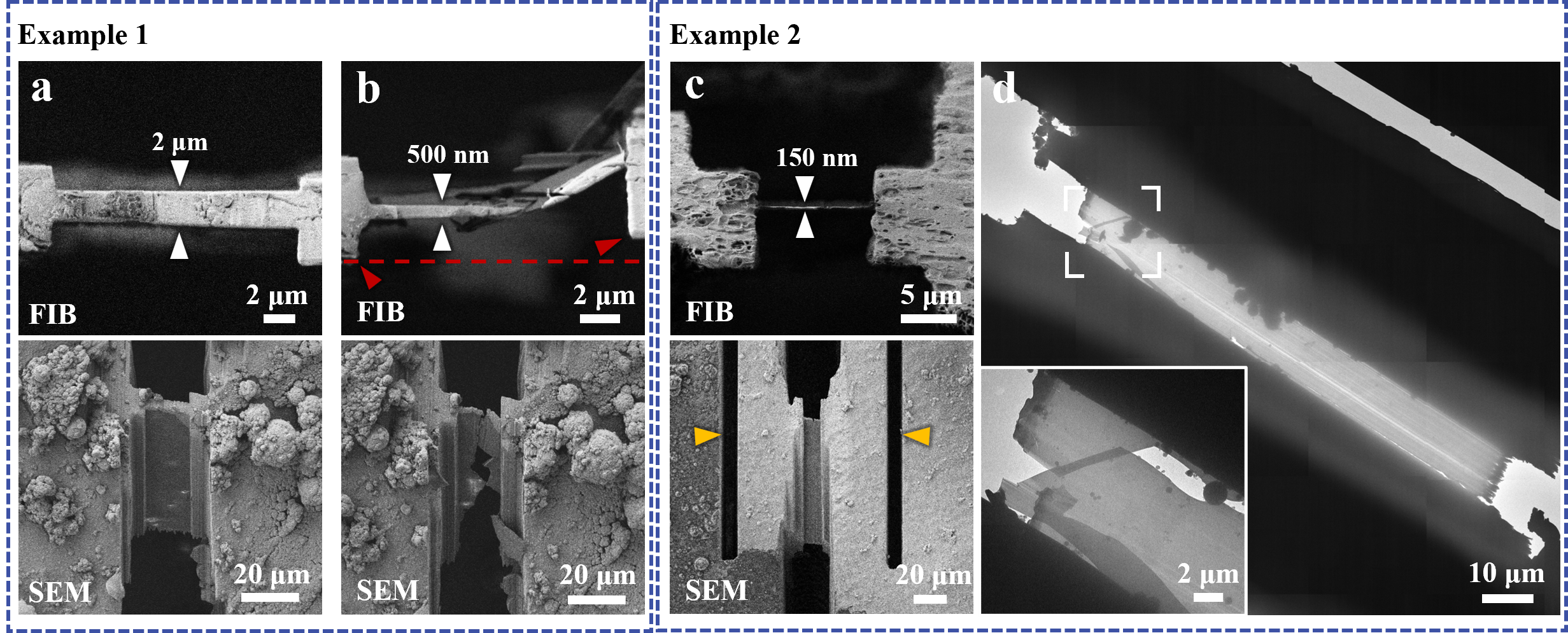


**Supplementary Figure 5. Examples of broken lamellae.** This figure demonstrates the importance of disconnecting one side of the lamella from the thick bulky sample to avoid breakage. **a,** A half-finished lamella of 2 μm thickness. **b,** The lamella shown in (**a**) broke when further milled to 500 nm thickness. The FIB view shows an offset between the left and right edges of the lamella (red arrows). **c,** Another lamella with 150 nm thickness. Two micro-expansion joints (yellow arrows) were created in order to release the inner stress^1^. The images under FIB view (top) and SEM view (bottom) are shown in panels (**a**) through (**c**). **d**, An image showing that the lamella was broken after being transferred to an electron microscope for cryoET data collection. The inset image shows the magnified area in the white box viewed from a different tilt angle. This indicates that the micro-expansion joint is often not sufficient for samples with large thicknesses. All images were acquired using Helios (FEI Company).


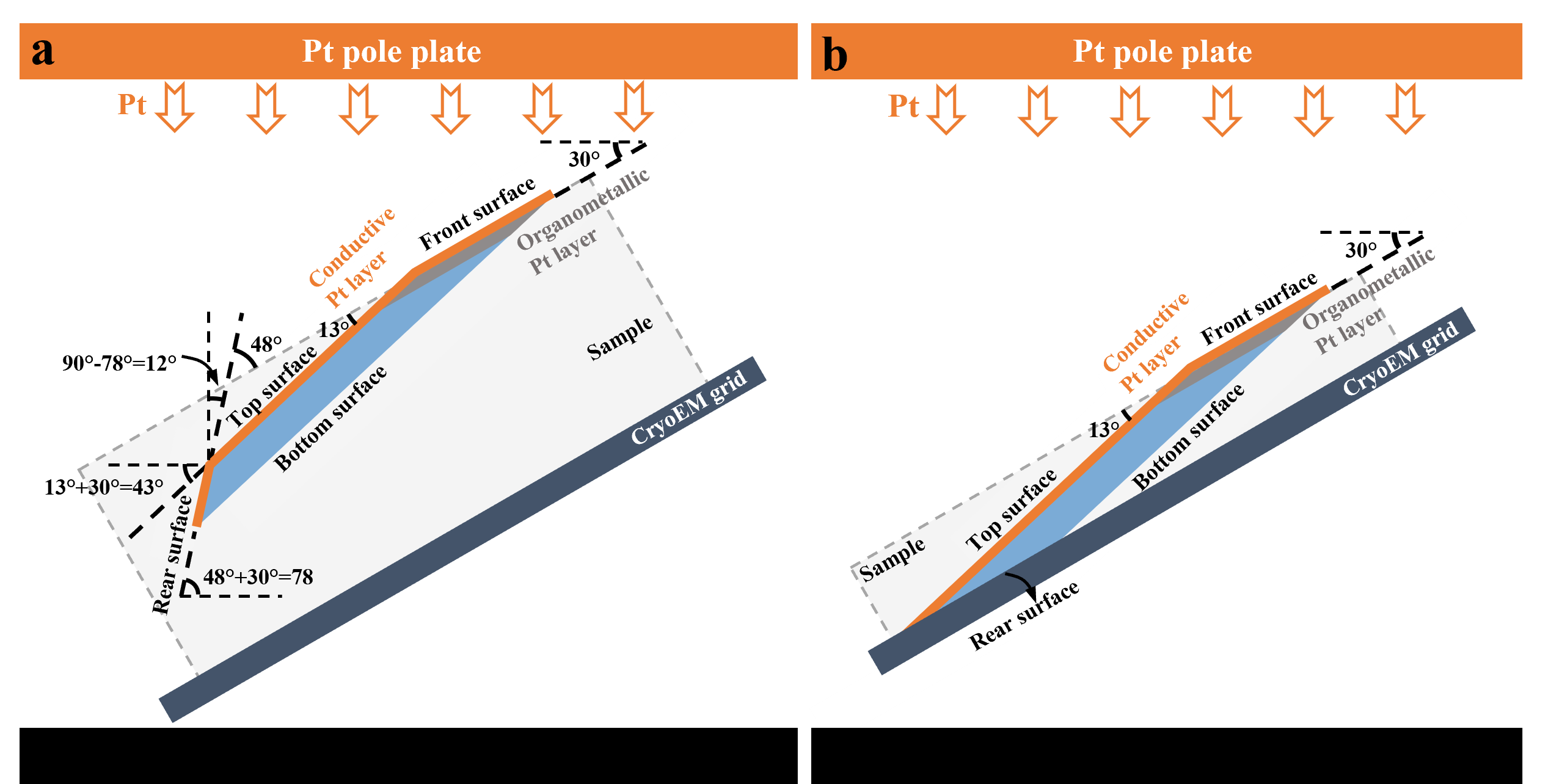


**Supplementary Figure 6. Schematic diagrams of the sputter coating performed after coarse milling.** **a.** The sputter coating and angular configuration of a sample inside the chamber for sputter coating, corresponding to **Fig. 3a**. The metallic Pt is deposited along the direction from the top Pt pole plate of the sputter device to the bottom. The cryoEM grid is mounted with a tilt angle of 30°. The front surface of the lamella is the top surface of the original sample and is parallel to the cryoEM grid plane. The angle between the top and the front surface of the lamella is determined by FIB direction 2, shown in **Fig. 3a** (13° in this case). The bottom surface of the lamella is parallel to the top surface. The angle between the rear surface and the front surface of the lamella is determined by FIB direction 1, shown in **Fig. 3a** (48° in this case). The angle between the rear surface of the lamella and the horizontal plane is 78° and must not exceed 90°, otherwise, the rear surface will be parallel to the direction of the Pt sputter coating or even shadowed, in which case it cannot be coated. **b,** The sputter coating and angular configuration of a sample after skipping the first sub-step of coarse milling inside the chamber for sputter coating, corresponding to **Fig. 3d**. The rear surface of the lamella is the bottom surface of the original sample, i.e., not facing the Pt source, and therefore, cannot be coated. Therefore, the milling step labeled with the number 6 shown in **Fig. 3a** is required to create a proper rear surface prior to sputter coating. All diagrams were drawn in side view. The metallic Pt element is colored orange, the organometallic Pt layer is colored dark grey, the lamella is colored light blue, the cryoEM grid is colored navy blue and the bulky sample is represented as a light gray volume.


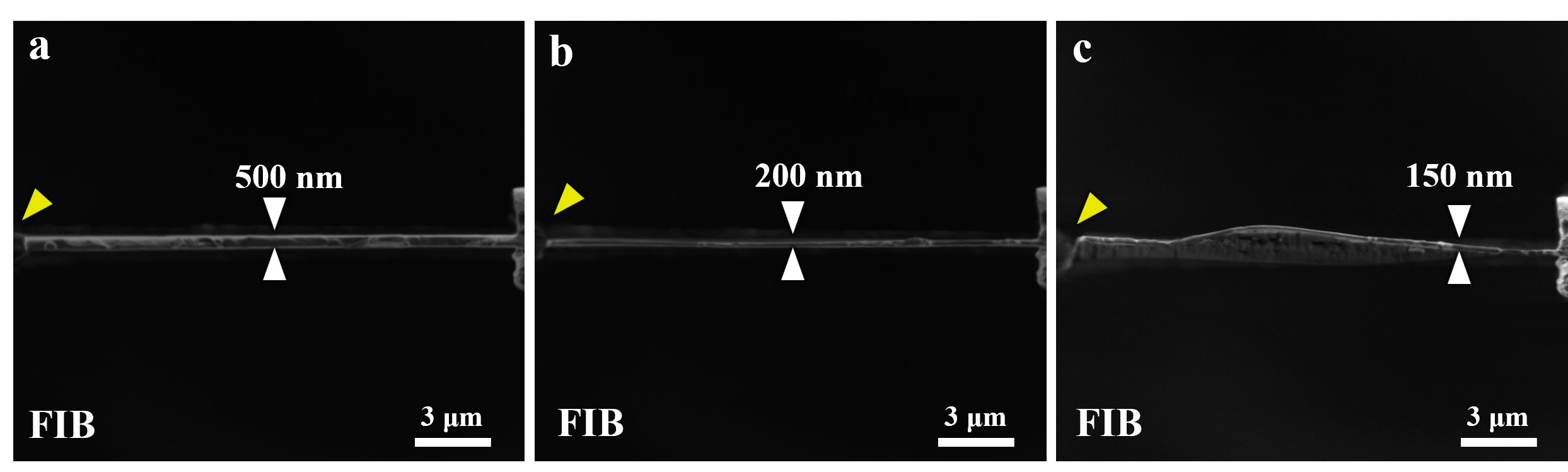


**Supplementary Figure 7. Bending of a lamella without the furrow-ridge structure.** The left side of the lamella is disconnected from the bulky sample (yellow arrows). The bending can be decomposed along two directions, lengthwise (perpendicular to the paper, or say, along the incident ion beam) and widthwise (horizontal on the paper and perpendicular to the incident ion beam). **a,** A lamella of ~500 nm thickness under FIB view, which was prepared from a lamella of 20 μm thickness. **b,** The lamella shown in **a** was further thinned to about 200 nm. Slight lengthwise bending is observed, represented as the fuzzy grey edge (generated by the far end of the lamella) on the upside of the lamella. **c,** The bending became more and more severe when the lamella was further thinned. Both the lengthwise and widthwise bending were observed, characterized by the wide fuzzy grey edge on the downside of the lamella and the distortion of the front edge of the lamella, respectively. The thinnest region of the lamella is approximately 150 nm. All images were acquired using Helios (FEI Company).


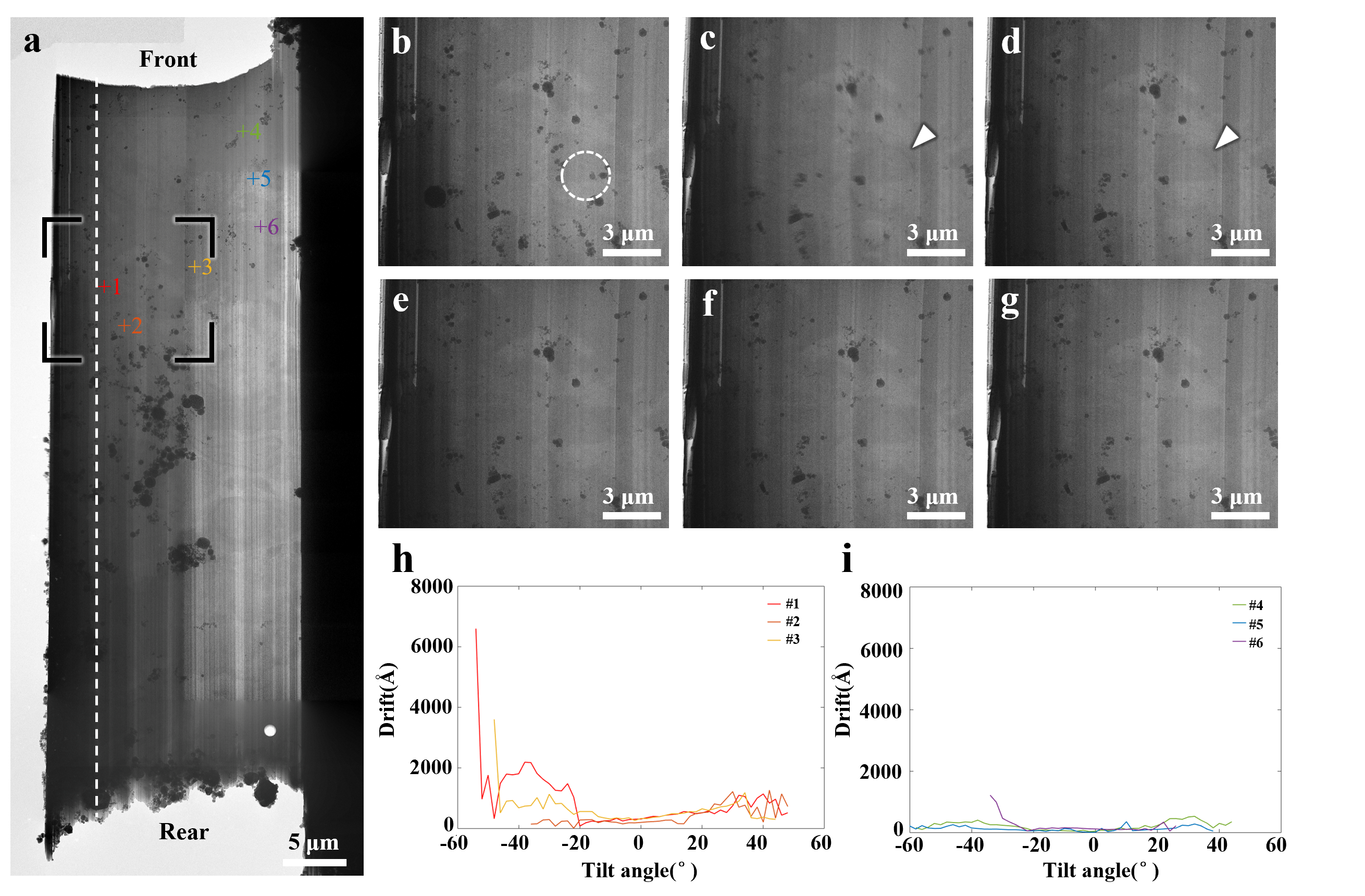


**Supplementary Figure 8. Observation of the charging and beam-induced motion of a lamella without the furrow-ridge structure. a,** A representative cryoEM image of a large lamella (20×60 μm). A region (black frame) was selected for observation. The front and rear surfaces of the lamella are marked. The areas used for tilt-series data collection are shown in colorful crosses labeled with numbers. The motion at the areas very close to the disconnected end of the lamella (on the left of the white dotted line) was so strong that the tracking at all tilt angles failed, hence no cryoET data was collected in these areas. **b,** A cryoEM image taken at the position indicated by the black frame in (**a**) before the exposure. The exposure was performed at a magnification of 33,000× and a dose of 2 e^−^/Å^2^. The illumination area of exposure is indicated by the white dashed circle. **c-g,** Continuous imaging of the same position as in (**a**) after the exposure (see also **Supplementary Movie 1**). An electron footprint^2^ (white arrow) and image distortion (see also **Supplementary Movie 1**) is built up by the charge accumulation around the illuminated area and gradually faded out during continuous imaging at low magnification. **h-i,** Curves of the measured drift (motion) against the tilt angles (relative to the plane of lamella) of cryoET data collection. The motion was measured in the regions labeled with colorful numbers in (**a**). The drift is the total shift of 8 dose-fractionation frames of a movie-mode micrograph acquired at a specific tilt angle. The total dose for a movie-mode micrograph is 2 e^−^/Å^2^. The beam-induced motion increases rapidly as getting closer to the disconnected end. For example, the motion in regions 1–3 is much larger than that in regions 4–6. Some large fluctuations of the curves in (**h-i)** are related to large errors of tracking. The tracking problem causes the following micrographs to be acquired in an unexpected area, which may have a different level of charge accumulation. Consequently, the motion measured on the corresponding micrograph might show an unexpected trend. Some data points at high tilt angles are missing due to the failure of tracking.


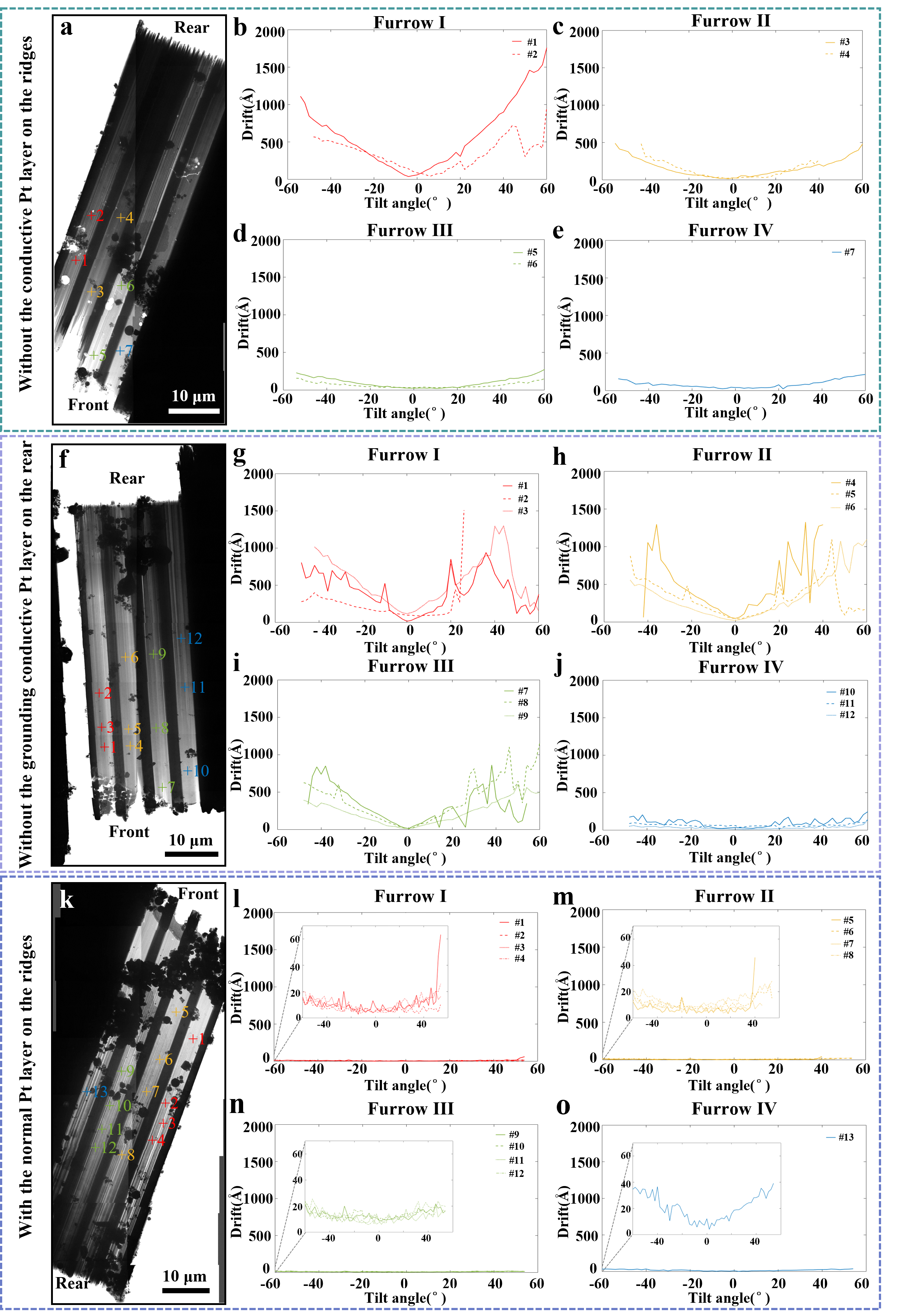


**Supplementary Figure 9.** **Beam-induced motion on the lamellae with or without conductive Pt layers on the ridges.** Three lamellae with different situations of conductive Pt layers were tested and compared**. a,** A low-magnification cryoEM image of a lamella without the conductive Pt layer on the ridges. **b-e,** Curves of the measured drift (motion) against the tilt angles (relative to the plane of lamella) of cryoET data collection. The motion was measured in several regions labeled with colorful numbers in (**a**). The drift is the total shift of 8 dose-fractionation frames of a movie-mode micrograph acquired at a specific tilt angle. The total dose for a movie-mode micrograph is 2 e^−^/Å^2^. The four furrows from the disconnected edge were defined as furrows I–IV in sequence. The colors, red, yellow, blue, and green, were used to indicate positions in furrows I–IV, respectively. For cryoET data collected in different furrows, drift was more severe as getting closer to the disconnected edge. **f,** A low-magnification cryoEM image of a lamella with a conductive, but not grounded, Pt layer on the ridges. **g-j,** Curves of the measured drift (motion) against the tilting angles of cryoET data collection. Similar to (**b**-**e**), the motion was measured in several regions labeled with colorful numbers in (**f**). The motion behaviors shown in (**g**-**j**) are similar to those of (**b**-**e**). Some severe fluctuations of the curves in (**b**-**e**) and (**g**-**j**) are related to large errors of tracking. The tracking problem causes the following micrographs to be acquired in an unexpected area, which may have a different level of charge accumulation. Consequently, the motion measured on the corresponding micrograph might show an unexpected trend. Some data points at high tilt angles are missing due to the failure of tracking. **k,** A low-magnification cryoEM image of a lamella with a normal Pt layer on the ridges. **l-o,** Curves of the measured drift (motion) against the tilting angles of cryoET data collection. The insets show the corresponding curves with amplified y-scale. Similar to (**b**-**e**), the motion was measured in several regions labeled with colorful numbers in (**k**). The motion measured in different furrows was significantly reduced, and similar in magnitude (below 60 Å).


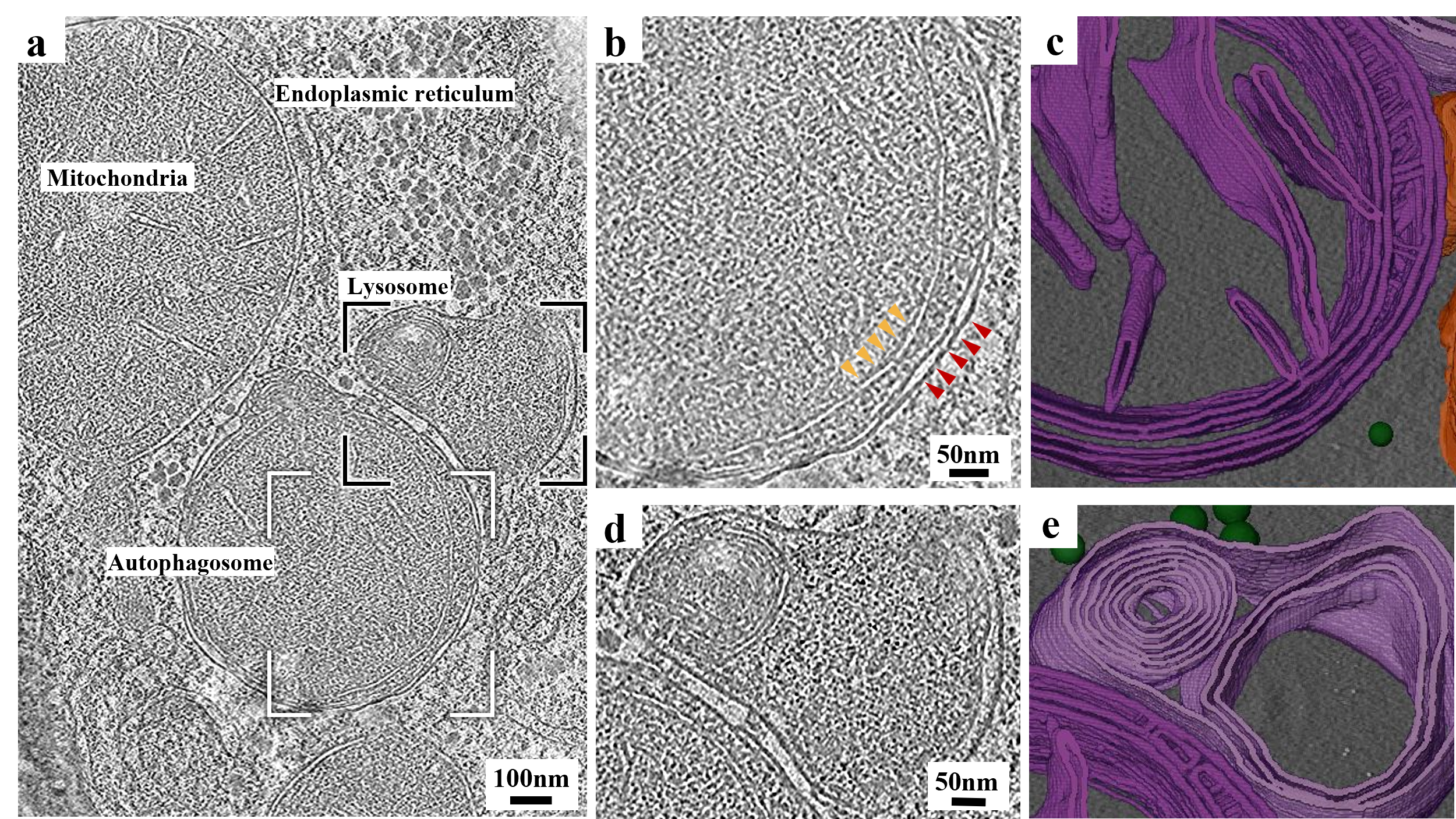


**Supplementary Figure 10. Tomogram of a liver tissue lamella.** A movie of the tomogram is shown in **Supplementary Movie 3**. **a,** Representative section view of a tomogram obtained from a lamella of healthy mouse liver tissue. **b,** Magnified view of an autophagosome labeled by the white box in (**a**). The double membrane of the autophagosome is indicated by red arrows. The double membrane of a mitochondrion wrapped inside the autophagosome is indicated by yellow arrows. **c,** 3D visualization of the membrane structure in (**b**). **d,** Magnified view of a lysosome labeled by the black box in (**a**). **e,** 3D visualization of the membrane structure in (**d**).

**Supplementary Tables**

**Supplementary Table 1. Typical milling settings and milling times without CSEI-based localization.** All values were based on samples of ~50 μm thickness.

|  | **Coarse milling** | | | | | | | **Fine milling** | |
| --- | --- | --- | --- | --- | --- | --- | --- | --- | --- |
|  | **Step 1*** | **Step 2** | **Step 3** | **Step 4** | **Step 5** | **Step 6** | **Step 7** | **Step 8** | **Step 9** |
| **FIB current** | 65 nA | 21 nA | 9.3 nA | 2.5 nA | 0.79 nA | 0.43 nA | 80 pA | 40 pA | 40 pA |
| **Lamella** [**thickness**](../../../../D:/wechat/WeChat%20Files/wxid_0fwg5uh1989k21/FileStorage/wechat/WeChat%20Files/wxid_0fwg5uh1989k21/FileStorage/File/wechat/WeChat%20Files/wxid_0fwg5uh1989k21/FileStorage/File/Dict/7.5.2.0/resultui/dict/%3Fkeyword=thickness) | 60 μm | 30 μm | 20 μm | 10 μm | 5 μm | 2.5 μm | 1 μm | 500 nm | 100–150 nm |
| **Pattern X size** | 80 μm | 50 μm | 40 μm | 30 μm | 25 μm | 23 μm | 20μm | 20 μm | 20 μm |
| **Typical time** | 50 min | 30 min | 20 min | 60 min | 60 min | 30 min | 50 min | 30 min | 60 min |

*In this step, the milling is performed at an angle of 48°.

**Supplementary Table 2. Typical milling settings and milling times with CSEI-based localization.** All values were based on samples of ~30 μm thickness.

|  | **Localization** | **Coarse milling** | | | | | | **Fine milling** | |
| --- | --- | --- | --- | --- | --- | --- | --- | --- | --- |
|  | **Step 1** | **Step 2** | **Step 3** | **Step 4** | **Step 5** | **Step 6** | **Step 7^※^** | **Step 8** | **Step 9** |
| **FIB current** | 5 nA | 9.3 nA | 2.5 nA | 0.79 nA | 0.43 nA | 80 pA | 40 pA | 40 pA | 40 pA |
| **Lamella** [**thickness**](../../../../D:/wechat/WeChat%20Files/wxid_0fwg5uh1989k21/FileStorage/wechat/WeChat%20Files/wxid_0fwg5uh1989k21/FileStorage/File/wechat/WeChat%20Files/wxid_0fwg5uh1989k21/FileStorage/File/Dict/7.5.2.0/resultui/dict/%3Fkeyword=thickness) | * | 20 μm | 10 μm | 5 μm | 2.5 μm | 1 μm | 1 μm | 500 nm | 100–150 nm |
| **Pattern X size** | 80 μm | 40 μm | 30 μm | 25 μm | 23 μm | 20 μm | 20μm | 20 μm | 20 μm |
| **Typical time** | * | 60 min | 40 min | 40 min | 30 min | 50 min | 10 min | 30 min | 60 min |

* The milling time and lamellae thickness for the CSEI-based localization process are variable with different samples.

**^※^** In this step, the rear of the lamellae is milled out at an angle of 48°.

**Supplementary Table 3. List of several typical examples.** Data on milled lamella size and number of tilt series collected on each lamella are shown for several samples with different initial thicknesses.

| Lamella no. | Sample thickness* | Lamella area | Number of tilt series collected**^※^** |
| --- | --- | --- | --- |
| Lamella 1 | 60 μm | 20 μm×53 μm | 24 |
| Lamella 2 | 35 μm | 20 μm×54 μm | 29 |
| Lamella 3 | 30 μm | 20 μm×60 μm | 22 |
| Lamella 4 | 52 μm | 20 μm×57 μm | 14 |

* Sample thickness (T) is measured using the cross-sectional height (H) of the liver tissue after the initial milling at an incidence angle of 48°(T=H×sin48°/sin52°).

**^※^**These values are not the maximum number of possible tilt series, since parts of the lamellae are covered with ice contamination and only the areas of interest have been collected.

**Supplementary Table 4. Comparison of efficiency and additional hardware requirement of different FIB milling methods**

| Method | Sample | Lamella thickness (nm) | Lamella area (µm^2^) | FIB milling time (h) | Milling efficiency (µm^2^/h) | Additional hardware requirement |
| --- | --- | --- | --- | --- | --- | --- |
| Cryo-lift out | C. elegans^3^ | 150 | ~8×7 | 10 | ~5.6 | Customized micromanipulator with a cryo-gripper tip, cryo-ultramicrotome |
|  | [Yeast](javascript:;)^4^ | 200–300 | ~6×4 | 4–6 | ~4–6 | Omniprobe cryo-micromanipulator |
| Waffle method | Yeast et al.^5^ | 100–200 | 12×15–20 | 2–3 | ~60–120 | Polished planchette hat, spacer ring (optional) |
|  | E. coli et al.^6^ | 200–250 | 12×12 | 2.5 | ~57.6 | Polished planchette hat, spacer ring (optional) |
| VHUT- cryoFIB | Liver tissue et al.^7^ | 200 | ~15×20 | 1.5 | ~200* | Cryo-ultramicrotome, customized cryo-carriers for HPF, cryo-transfer shuttle and cryo-holder tip |
| This method | Liver tissue | 100–150 | ~20×50 | 6 | ~167 | None |

* The time used by cryo-ultramicrotomy processing wasn’t counted, which would significantly reduce the overall efficiency.

**References**

1. Wolff, G. *et al*. Mind the gap: Micro-expansion joints drastically decrease the bending of FIB-milled cryo-lamellae. *J Struct Biol* **208**, 107389 (2019).

2. Brink J, Sherman MB, Berriman J, Chiu W. Evaluation of charging on macromolecules in electron cryomicroscopy. *Ultramicroscopy* **72**, 41-52 (1998).

3. Schaffer M*, et al.* A cryo-FIB lift-out technique enables molecular-resolution cryo-ET within native Caenorhabditis elegans tissue. *Nat Methods* **16**, 757-762 (2019).

4. Parmenter CD, Nizamudeen ZA. Cryo-FIB-lift-out: practically impossible to practical reality. *J Microsc* **281**, 157-174 (2021).

5. Klykov O*, et al.* In situ cryo-FIB/SEM Specimen Preparation Using the Waffle Method. *Bio Protoc* **12**, (2022).

6. Kelley K, et al. Waffle Method: A general and flexible approach for improving throughput in FIB-milling. Nat Commun 13, 1857 (2022).

7. Zhang J*, et al.* VHUT-cryo-FIB, a method to fabricate frozen hydrated lamellae from tissue specimens for in situ cryo-electron tomography. *J Struct Biol* **213**, 107763 (2021).
